# Distinct microbial hydrogen and reductant disposal pathways explain interbreed variations in ruminant methane yield

**DOI:** 10.1093/ismejo/wrad016

**Published:** 2024-01-10

**Authors:** Qiushuang Li, Zhiyuan Ma, Jiabin Huo, Xiumin Zhang, Rong Wang, Shizhe Zhang, Jinzhen Jiao, Xiyang Dong, Peter H Janssen, Emilio M Ungerfeld, Chris Greening, Zhiliang Tan, Min Wang

**Affiliations:** Key Laboratory for Agro-Ecological Processes in Subtropical Region, Institute of Subtropical Agriculture, Chinese Academy of Sciences, Changsha, Hunan 410125, China; University of Chinese Academy of Sciences, Beijing 100049, China; College of Pastoral Agriculture Science and Technology, Lanzhou University, Lanzhou 730000, China; Key Laboratory for Agro-Ecological Processes in Subtropical Region, Institute of Subtropical Agriculture, Chinese Academy of Sciences, Changsha, Hunan 410125, China; Key Laboratory for Agro-Ecological Processes in Subtropical Region, Institute of Subtropical Agriculture, Chinese Academy of Sciences, Changsha, Hunan 410125, China; Key Laboratory for Agro-Ecological Processes in Subtropical Region, Institute of Subtropical Agriculture, Chinese Academy of Sciences, Changsha, Hunan 410125, China; Key Laboratory for Agro-Ecological Processes in Subtropical Region, Institute of Subtropical Agriculture, Chinese Academy of Sciences, Changsha, Hunan 410125, China; Key Laboratory for Agro-Ecological Processes in Subtropical Region, Institute of Subtropical Agriculture, Chinese Academy of Sciences, Changsha, Hunan 410125, China; Key Laboratory of Marine Genetic Resources, Third Institute of Oceanography, Ministry of Natural Resources, Xiamen 361005, China; AgResearch Limited, Grasslands Research Centre, Palmerston North, Private Bag 11008, New Zealand; Centro Regional de Investigación Carillanca, Instituto de Investigaciones Agropecuarias (INIA), Temuco, Vilcún 4880000, Chile; Department of Microbiology, Biomedicine Discovery Institute, Monash University, Clayton, VIC 3800, Australia; Key Laboratory for Agro-Ecological Processes in Subtropical Region, Institute of Subtropical Agriculture, Chinese Academy of Sciences, Changsha, Hunan 410125, China; University of Chinese Academy of Sciences, Beijing 100049, China; Key Laboratory for Agro-Ecological Processes in Subtropical Region, Institute of Subtropical Agriculture, Chinese Academy of Sciences, Changsha, Hunan 410125, China; University of Chinese Academy of Sciences, Beijing 100049, China

**Keywords:** rumen fermentation, methanogenesis, electron transfer, molecular hydrogen, hydrogenase, methane emissions

## Abstract

Ruminants are essential for global food security, but these are major sources of the greenhouse gas methane. Methane yield is controlled by the cycling of molecular hydrogen (H_2_), which is produced during carbohydrate fermentation and is consumed by methanogenic, acetogenic, and respiratory microorganisms. However, we lack a holistic understanding of the mediators and pathways of H_2_ metabolism and how this varies between ruminants with different methane-emitting phenotypes. Here, we used metagenomic, metatranscriptomic, metabolomics, and biochemical approaches to compare H_2_ cycling and reductant disposal pathways between low-methane-emitting Holstein and high-methane-emitting Jersey dairy cattle. The Holstein rumen microbiota had a greater capacity for reductant disposal via electron transfer for amino acid synthesis and propionate production, catalyzed by enzymes such as glutamate synthase and lactate dehydrogenase, and expressed uptake [NiFe]-hydrogenases to use H_2_ to support sulfate and nitrate respiration, leading to enhanced coupling of H_2_ cycling with less expelled methane. The Jersey rumen microbiome had a greater proportion of reductant disposal via H_2_ production catalyzed by fermentative hydrogenases encoded by *Clostridia*, with H_2_ mainly taken up through methanogenesis via methanogenic [NiFe]-hydrogenases and acetogenesis via [FeFe]-hydrogenases, resulting in enhanced methane and acetate production. Such enhancement of electron incorporation for metabolite synthesis with reduced methanogenesis was further supported by two *in vitro* measurements of microbiome activities, metabolites, and public global microbiome data of low- and high-methane-emitting beef cattle and sheep. Overall, this study highlights the importance of promoting alternative H_2_ consumption and reductant disposal pathways for synthesizing host-beneficial metabolites and reducing methane production in ruminants.

## Introduction

Ruminant livestock are essential for global food security. Ruminants harbor a complex rumen microbial ecosystem that transforms fibrous feed into nutrients the animal host uses to form high-quality products such as meat and milk, while producing methane (CH_4_) as a natural product of the anaerobic microbial fermentation in rumen [1, 2]. About 30% of global anthropogenic CH_4_ emissions originate from livestock production, of which 88% comes from enteric fermentation [3, 4]. Moreover, enteric CH_4_ release results in considerable energy loss for the host animal, ranging from 2 % to12% of total energy intake [5]. Strategies for enteric CH_4_ mitigation include methanogenesis inhibitors [6], selective breeding [7], dietary modulation [8], the promotion of alternative hydrogen (H_2_) sinks [9], and vaccines [10]. These strategies depend on reducing the activity of CH_4_-forming microbes and redirecting the substrates used for ruminal methanogenesis into absorbable metabolites such as propionate.

Molecular H_2_, the main substrate for ruminal methanogenesis, is primarily produced during carbohydrate fermentation [11]. After the hydrolysis of complex feed carbohydrates, the resulting monosaccharides are fermented through a series of oxidation steps, resulting in the formation of pyruvate, which can be further oxidized to the volatile fatty acid (VFA) acetate. The electrons liberated during this process are transferred to cellular electron carriers such as NAD^+^, NADP^+^, and ferredoxin [12]. The resultant reductant can be disposed of via electron transfer reactions to oxidized metabolic intermediates of the fermentation pathways to produce more reduced products, such as the VFAs propionate and butyrate, the amino acid glutamate, or lactate [13-16]. These compounds, as well as acetate, are absorbed and used as nutrients by the host animal. Most fermentative microorganisms also use hydrogenases to couple the reoxidation of nicotinamide adenine dinucleotide (NADH) and reduced ferredoxin to the production of H_2_. The H_2_ dissolves in the rumen liquid and can be used as an energy source by hydrogenotrophic microorganisms, including methanogenic archaea [15, 17-20]. Sufficiently low dissolved H_2_ concentrations (<50 μM) must be maintained for fermentation to be thermodynamically favorable [21, 22], which is achieved through the coupling of fermentative H_2_ production with various H_2_ consumption pathways. The fermentation product spectrum is then determined by the amount of acetate formed and the disposal of the resultant electrons as H_2_, CH_4_, propionate, butyrate, and other reduced products. However, the factors involved in controlling reductant disposal and H_2_ metabolism that maintain a low H_2_ concentration and methanogenesis in the rumen microbial ecosystem have received insufficient investigation [23].

Although most H_2_ is used by methanogens for methanogenesis [21], various hydrogenotrophic bacteria compete with methanogens for H_2_ [24]. Reductive acetogenesis, i.e. the reduction of CO_2_ with H_2_ to acetate, occurs in the gastrointestinal tract of many animals. However, the process typically occurs at lower rates than methanogenesis in ruminants, because that acetogens have a lower affinity for H_2_ and harvest less energy [21, 25]. Bacteria that use other electron acceptors, such as nitrate and sulfate, can thermodynamically outcompete methanogenesis [26]. However, the low availability of these electron acceptors in the rumen usually limits their significance for anaerobic H_2_ use [27], unless these acceptors are supplemented into the diet at a safe dose [28]. There is also increasing evidence of substantial populations of hydrogenotrophic fumarate-reducing bacteria in the rumen [24, 29]. Generally, these pathways are assumed to be minor ones, but can still be regarded as alternative pathways of H_2_ incorporation.

Variations in ruminant CH_4_ production are strongly correlated with the composition and function of rumen microbiota [30-32]. Currently, we lack a strong understanding of how these differences relate to the pathways and mediators of H_2_ cycling and reductant disposal. Here, we postulate that contrasting CH_4_-emitting phenotypes are associated with distinct processes of reductant disposal via electron transfer and H_2_ metabolism. We studied two dairy breeds (i.e. Holstein and Jersey dairy cows) that differed in their CH_4_ emissions and integrated *in vivo* and *in vitro* experiments with multi-omics approaches to explore the metabolism of their rumen microbiota. The study revealed that the rumen microbiota of low-CH_4_-emitting Holstein cows differed in composition, capabilities, and activities compared with the microbiota of the high-CH_4_-emitting Jerseys. The analysis of global microbiomes of beef cattle and sheep also further supported that reduced CH_4_ emissions are associated with enhanced electron incorporation into metabolites used by the host instead of methanogenesis. Collectively, these results suggest that low-CH_4−_emitting rumen microbiota redirect H_2_ and reductant flux toward fatty acids and microbial biomass production, potentially as a beneficial evolutionary response to maximize energy conservation.

## Results

### Jersey and Holstein dairy cows differ in their methane-emitting phenotypes and rumen microbial communities

**Figure 1:**
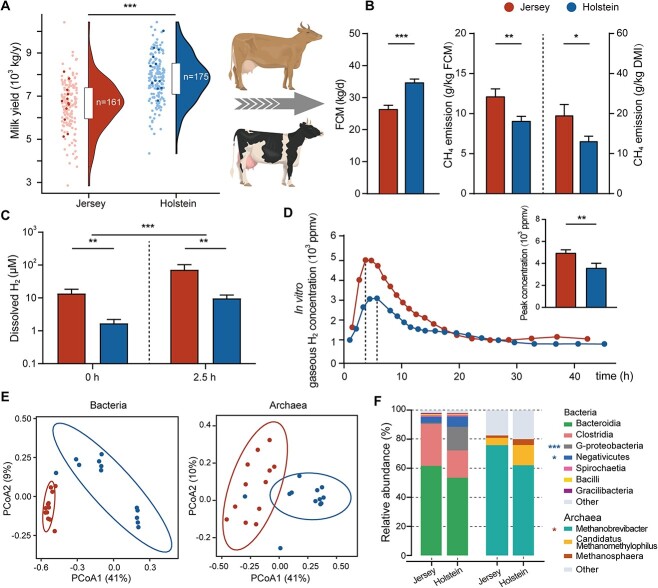
Production variables of Jersey (red) or Holstein (blue) dairy cows and their distinct rumen H_2_ dynamics and prokaryotic communities; (A) distribution of milk production of selected 24 cows (darker points) in Jersey (*n* = 161) or Holstein (*n* = 175) dairy herd; (B) the intensity and yield of CH_4_ emissions correct for FCM production and DMI, respectively; (C) ruminal H_2_ concentrations at +0 and + 2.5 h relative to the morning feeding; (D) the time course of H_2_ gas and peak concentrations for *In Vitro* Experiment 1 with feed incubated with Jersey or Holstein rumen inocula; (E) beta diversity of bacterial and archaeal communities based on 16S rRNA gene ASVs (Bray–Curtis dissimilarity matrix, PERMANOVA, bacteria: *P* = .001, *R*^2^ = 0.31; archaea: *P* = .001, *R*^2^ = 0.33); (F) class-level bacterial and genus-level archaeal community composition based on 16S rRNA gene amplicon sequence; DM, dry matter; G-proteobacteria, *Gammaproteobacteria*; data with error bars are expressed as mean ± standard error; ^*^P < .05, ^*^^*^P < .01, ^*^^*^^*^P < .001, *n* = 12.

We selected 12 Jersey and 12 Holstein dairy cows representative of their herds, which were fed the same diet (Supplementary Table S1), based on their first lactation 305-day milk yield (Supplementary Table S2). For each herd, milk production of the selected cows was evenly distributed on both sides of the median milk yield (161 Jersey dairy cows and 175 Holstein dairy cows, Fig. 1A). At 220 days in milk of their second lactation, the Holstein dairy cows exhibited higher dry matter intake (DMI; +34.1%, *P* < .001) and fat-corrected milk yield (FCM; +31.7%, *P* < .001; Fig. 1B, Supplementary Table S3). The Holstein dairy cows exhibited lower enteric CH_4_ intensity (i.e. CH_4_ emissions expressed as g/kg FCM; −25.4%, *P* < .01) and lower CH_4_ yield (i.e. CH_4_ emissions per kg DMI; Fig. 1B, Supplementary Table S4 −35.0%, *P* < .001) than the Jersey dairy cows.

Dissolved H_2_ (dH_2_) concentrations are determined by the balance between H_2_ production and consumption pathways in the rumen microbial ecosystem [11, 15]. Holstein dairy cows exhibited lower rumen dH_2_ concentrations than Jersey dairy cows before and after feeding (Fig. 1C, *P* < .01). Furthermore, *In Vitro* Experiment 1 indicated that rumen contents from Holstein cows produced lower levels of gaseous H_2_ (gH_2_) compared to rumen contents from Jersey cows (Fig. 1D, *P* < .01). Together, these results suggest underlying differences in H_2_ transactions between Holstein and Jersey rumen microbiota.

The composition and diversity of the rumen microbiota differed between the two breeds. We obtained 4465 bacterial amplicon sequence variants (ASVs) and 314 archaeal ASV across the 24 rumen content samples through 16S ribosomal RNA (rRNA) gene amplicon sequencing (Supplementary Fig. S1). Although no differences were observed in 16S rRNA gene copies of bacteria and methanogens (*P* > .10; Supplementary Fig. S2), the evenness of the bacterial and archaeal communities was lower in the Holstein rumen samples (*P* < .05; Supplementary Fig. S3). Based on Bray–Curtis beta diversity, the composition of bacterial and archaeal communities in the Holstein rumen microbiome was distinct from their counterparts in the Jersey rumen microbiome (permutational multivariate ANOVA [PERMANOVA]; bacteria: *P* = .001, *R*^2^ = 0.31; archaea: *P* = .001, *R*^2^ = 0.33, Fig. 1E). The Holstein rumen microbiome was enriched in two bacterial classes: *Gammaproteobacteria* (*P* < .001; 0.83% vs. 16.3% for Jersey and Holstein, respectively) and *Negativicutes* (*P* < .05; 4.2% vs. 7.1%, Fig. 1F and Supplementary File S1), and 76 bacterial genera were found to exhibit significant differential abundances (Supplementary Fig. S4 and Supplementary File S1). In the archaeal community, the *Methanobrevibacter* was more dominant in the Jersey rumen microbiome (*P* < .05, 75.9% vs. 62.9%; Fig. 1F and Supplementary File S2). Furthermore, qPCR analysis indicated that Jersey rumen samples had more 16S rRNA gene copies of *Methanobrevibacter* than Holstein (5.4-fold higher, *P* < .01, Supplementary Fig. S2). In contrast, methylotrophic methanogens from the *Methanosphaera* and *Candidatus Methanomethylophilus* were enriched in the Holstein rumen samples (Fig. 1F).

### Jersey and Holstein rumen samples differ in metabolite levels

**Figure 2:**
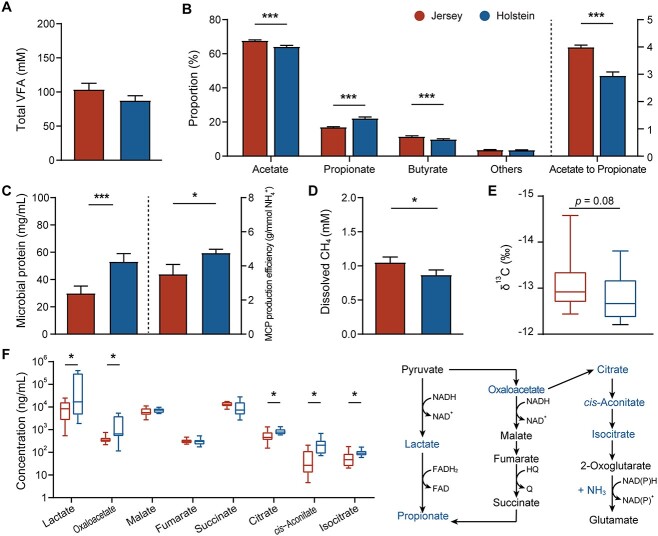
The distinct microbiome metabolome in the rumen of Jersey (red) and Holstein (blue) dairy cows; (A) VFA concentrations; (B) molar proportion of individual VFAs; (C) concentrations and estimated production efficiency of MCP; (D) dCH_4_ concentrations; (E) fractionation of stable carbon isotopes in acetate; (F) proposed carbon metabolism associated with propionate and glutamate synthesis; the blue font indicated compounds significantly increased in the Holstein rumen at *P* < .05; ^*^P < .05, ^*^^*^P < .01, ^*^^*^^*^P < .001, *n* = 12.

Although there were no statistically significant differences in the overall VFA concentrations (*P* = .34; Fig. 2A), Holstein rumen samples contained a greater molar proportion of propionate (*P <* .001; Fig. 2B; Supplementary Table S5) and lower acetate to propionate ratios (*P <* .001; Fig. 2B; 2.95 vs. 4.00 for Jersey and Holstein, respectively). In contrast, Jersey rumen samples contained greater molar proportions of acetate (*P <* .001) and butyrate (*P <* .001; Fig. 2B; Supplementary Table S5). Our results also show that the Holstein rumen contained a greater microbial protein (MCP) concentration in the rumen fluid (*P <* .001; Fig. 2C; Supplementary Table S5) and higher fluid MCP to NH_4_^+^ concentration ratio (*P <* .05; Fig. 2C). These results indicated that the Holstein dairy cows had a phenotype typical of low-CH_4_ emitters compared with Jerseys, with lower acetate to propionate ratios and greater MCP synthesis in rumen fluid.

We used targeted metabolomics of central carbon metabolism to measure the concentrations of 30 metabolic intermediates of propionate production and glutamate synthesis, which are the important pathways of reductant disposal. There were differences in the concentrations of lactate, oxaloacetate, citrate, *cis*-aconitate, and isocitrate, which were all greater in the Holstein rumen (*P* < .05; Fig. 2F; Supplementary File S3). Our metabolite results indicated greater glutamate synthesis, consistent with glutamate being the major donor for synthesizing other amino acids [33]. These findings and the measured fermentation products were integrated into the model in Fig. 2F, suggesting that the Holstein rumen microbiota increased propionate and MCP production through the acrylate pathway and reductive amination, respectively.

Dissolved CH_4_ (dCH_4_) concentrations were greater in the rumen of Jersey cows (*P* < .05; Fig. 2D), which is consistent with their greater enteric CH_4_ yield (Fig. 1B). Isotopic fractionation can be used to distinguish the acetate produced from H_2_ and CO_2_ by reductive acetogenesis from the acetate resulting from carbohydrate oxidation [34]; acetate formed from H_2_ and CO_2_ through the reductive Wood–Ljungdahl pathway has a strong fractionation (i.e. greater in absolute value: −55‰ to −60‰) [35-37], whereas isotopic fractionation associated with acetate fermentation via carbohydrate fermentation has been found to be weaker (i.e. smaller in absolute value: −5‰) [38-41]. Acetate from the rumen of Jersey cows tended to have a lower δ^13^C (stronger fractionation) than did acetate from Holstein cows (*P* = .08; −13.1‰ vs. −12.8‰; Fig. 2E). Lower enrichment of δ^13^C in acetate suggests that reductive acetogenesis is potentially enhanced in the rumen of Jersey cows compared with Holstein. Overall, these results indicate that Jersey rumen microbiota utilizes more H_2_ through methanogenesis and perhaps reductive acetogenesis to a minor extent, consistent with a larger molar proportion of acetate and greater *in vitro* and *in vivo* dH_2_ concentration in these cattle.

### Distinct composition and function of Jersey and Holstein rumen microbiomes

Metagenomes and metatranscriptomes were sequenced to better understand the microorganisms and processes controlling the observed differences in rumen fermentation (Supplementary File S4). Following quality control and removal of reads assigned to the host, a total of 382 Gb and 146 Gb of paired-end sequencing data were generated from the metagenomes (15.9 ± 1.8 Gb; mean per sample ±  standard error of mean (SEM)) and metatranscriptomes (6.1 ± 2.9 Gb; mean per sample ± SEM), respectively. In agreement with our amplicon sequencing results, taxonomic profiling of metagenomes and metatranscriptomes indicated that the composition of bacterial and methanogen communities was distinct between Holstein and Jersey rumen microbiomes (Supplementary Figs S5 and S6).

**Figure 3:**
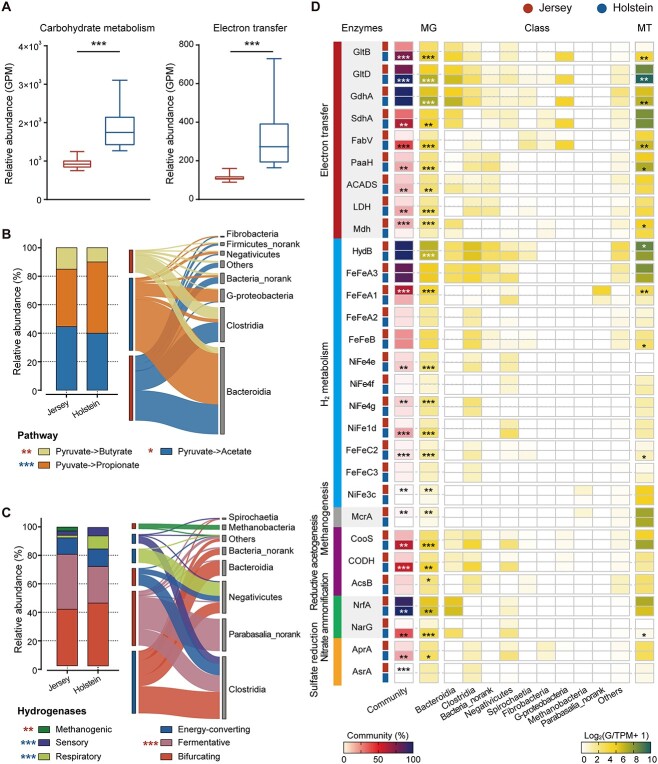
Distinct microbiome function in the rumen of Jersey (red) and Holstein (blue) dairy cows; (A) relative abundance of genes belonging to carbohydrate metabolism and reductant disposal via electron transfer during carbohydrate degradation and glutamate synthesis; box and whisker plots with red and blue dots correspond to Jersey and Holstein dairy cows, respectively; (B) the relative abundance of genes involved in acetate, propionate, and butyrate production, and their enrichment in various prokaryotic classes; (C) the relative abundance of hydrogenases of different function and their enrichment in various prokaryotic classes; (D) key metabolic genes detected using homology-based searches in metagenomes (MG) and transcripts in metatranscriptomes (MT), and their phylogenetic distribution at class-level of the most abundant prokaryotes; only genes with an average relative abundance >1% are shown; the proportion of community members in each metagenome predicted to encode each gene was based on short reads; hits were normalized to gene length and single-copy ribosomal marker genes using SingleM; genes are involved in reductant disposal via electron transfer during carbohydrate degradation and glutamate synthesis, molecular H_2_ metabolism, methanogenesis, reductive acetogenesis, nitrate ammonification, and sulfate reduction; the color of asterisks in B and C indicates in which breed the inferred microbial activity is greater; G-proteobacteria, *Gammaproteobacteria*; taxonomy assignment is based on NCBI-NR (October 2018; ~550 M sequences); whiskers represent the lowest and highest values; ^*^P < .05, ^*^^*^P < .01, ^*^^*^^*^P < .001, *n* = 12.

The metabolic capacity of the rumen microbiome was first annotated using the Kyoto Encyclopedia of Genes and Genomes (KEGG) database [42]. Principal coordinate analysis of all KEGG orthology (KO) genes showed that the Jersey and Holstein rumens selected for different metabolic functions (*P* < .001, *R*^2^ = 0.31; Supplementary Fig. S7A), with the most abundant category, “carbohydrate metabolism pathways,” making up a significantly larger proportion in the Holstein rumen (*P* < .001; Supplementary Fig. S7B and Supplementary File S5). The abundance and transcripts of genes involved in carbohydrate metabolism indicated that the Holstein rumen microbiome had greater carbohydrate metabolism capacity and activity (*P* < .001; Fig. 3A; Supplementary File S5), which is consistent with the greater relative abundance and transcripts of total carbohydrate-active enzymes (CAZymes) such as *β*-xylosidase GH43 (*P* < .05; Supplementary Fig. S8; Supplementary File S6). These results highlight that the Holstein rumen microbiome has a greater capacity for carbohydrate metabolism.

Furthermore, the Holstein rumen microbiome was enriched with a greater proportion of genes for enzymes potentially producing propionate from pyruvate, which were mainly assigned to the classes *Bacterioidia* and *Gammaproteobacteria* (*P* < .05; Fig. 3B). In contrast, the Jersey rumen microbiome encoded a greater proportion of enzymes that produce acetate and butyrate from pyruvate, which were mainly assigned to *Bacterioidia* and *Clostridia* (*P* < .05; Fig. 3B). These results indicate that, although acetate is the primary product of ruminal fermentation in both breeds, the Holstein rumen microbiome shifted the partitioning of fermentation intermediates toward propionate.

During the fermentation of carbohydrates in the rumen, butyrate, propionate, and glutamate synthesis can be associated with different pathways of reductant disposals. We therefore measured the levels and transcripts of marker genes related to reductant disposal pathways (Supplementary File S4). The Holstein rumen microbiome contained a greater abundance of total marker genes for electron transfer reactions (*P* < .001; 2.9-fold higher; Fig. 3A). There was a higher proportion of the genes *gltB*, *gltD*, *gdhA* (encoding glutamate synthase; *P* < .001), *sdhA*/*frdA* (encoding fumarate reductase involved in propionate formation via the succinate pathway), *paaH* and *fabV* (encoding acetoacetyl-CoA reductase and crotonoyl-CoA reductase associated with butyrate production), *ldh* (encoding lactate dehydrogenase), and *ACADS* (encoding short-chain-specific acyl-CoA dehydrogenases producing propionate or butyrate) (*P* < .01; Fig. 3D). The increased abundance of *ACADS* may indicate a significant role in propionate formation via the acrylate pathway in the Holstein rumen. In agreement with these metagenomic results, the metatranscriptomic analysis showed that the Holstein rumen microbiome produced more transcripts of *gltB*, *gltD*, *gdhA*, *paaH, fabV*, and *ldh* (*P* < .05; Fig. 3D). To the best of our knowledge, these findings are the first to show that the Holstein rumen microbiome has an enhanced ability to dispose of reductant via electron transfer to form more reduced organic products compared with Jersey cows.

After screening for genes encoding for catalytic subunits of H_2_-producing and H_2_-consuming enzymes in the assembled contigs, we annotated 1384 hydrogenases, which included 1086 [FeFe]-hydrogenases, 294 [NiFe]-hydrogenases, and 4 [Fe]-hydrogenases. Phylogenetic analysis also validated the presence and reliability of the identified hydrogenase sequences (Supplementary Figs S9–S11). Diverse hydrogenases were present in the rumen microbiome and assigned to 87 genera, including *Bacteroides*, *Clostridium*, *Methanobrevibacter*, and *Ruminococcus* (Supplementary File S7). The Holstein rumen microbiome had a greater abundance of genes encoding for sensory and respiratory hydrogenases, such as group C2 [FeFe]-hydrogenase (*P* < .001; Fig. 3C and D; 3.2-fold higher) and group 1d [NiFe]-hydrogenase, which were, respectively, assigned to *Clostridia* and *Negativicutes* (Supplementary File S7). The Jersey rumen microbiome had a greater abundance of genes encoding for fermentative hydrogenases (*P* < .001; Fig. 3C), mainly group A1 [FeFe]-hydrogenases assigned to protists (*P* < .001; Fig. 3D and Supplementary File S7; 3.0-fold higher), and methanogenic hydrogenases such as the F_420_-reducing group 3a [NiFe]-hydrogenase encoded by *Methanobrevibacter* (*P* < .01; Fig. 3D and Supplementary File S7; 3.4-fold higher). Metatranscriptomic analysis indicated that the Jersey rumen microbiome expressed fermentative group A1 and electron-bifurcating group A3 [FeFe]-hydrogenases (*P* = .06) at higher levels, which are the primary catalysts of H_2_ production in ruminants [24]. The higher relative abundance of genes encoding H_2_-producing hydrogenases in the Jersey rumen microbiome agrees with the higher dH_2_ concentrations in their rumen fluid. These results indicate that the fermentative species in the Jersey rumen microbiome may have an enhanced ability to dispose of reductants as H_2_, resulting in higher H_2_ concentrations that may enhance hydrogenotrophic processes.

We further analyzed signature genes that support hydrogenotrophic growth, including methanogenesis, acetogenesis, and respiratory use of nitrate and sulfur compounds. The Holstein rumen microbiome encoded higher levels of nitrate reductase (*narG*; with similar results in the parallel metatranscriptomic analysis) involved in nitrate reduction to nitrite (*P* < .001) and nitrite reductase (*nrfA*) involved in nitrite reduction to ammonium (*P* < .01; Fig. 3D), indicating an enhancement of nitrate ammonification, although their transcript numbers were low. Moreover, the Holstein rumen microbiome also had a greater abundance of genes for adenylylsulfate reductase (*aprA*) (*P* < .05; Fig. 3D). In contrast, the Jersey rumen microbiome had a greater abundance of *mcrA* (*P* < .05; Fig. 3D; Supplementary File S8), encoding for methyl-CoM reductase, catalyzing the last step of CH_4_ formation, and mainly affiliated with *Methanobrevibacter* (Supplementary File S7). Furthermore, around 28% *mcrA* reads in the Holstein rumen were contributed by methylotrophic members of the order *Methanomassilicoccales*, including sequences annotated as *Candidatus Methanomethylophilus* (Supplementary File S7 and Supplementary Fig. S12), compared to only 6% in the Jersey rumen. Additionally, several key enzymes associated with methylotrophic methanogenesis exhibited higher relative abundance and transcript levels in the Holstein rumen compared to Jersey (Supplementary Fig. S13). This indicates a reduced relative significance of methanogenesis from H_2_ plus CO_2_ in the Holstein rumen. The Jersey rumen microbiome had a greater abundance of *ascB* (*P* < .05; Fig. 3D; 1.2-fold higher), a signature gene for reductive acetogenesis through the Wood–Ljungdahl pathway, and was mainly assigned to *Clostridia*. Together, these results suggest that the Holstein rumen microbiome facilitates more H_2_ uptake through various respiratory processes, whereas the Jersey rumen microbiome appears to favor more H_2_ utilization through hydrogenotrophic methanogenesis and reductive acetogenesis.

### Prokaryotic classes capable of reductant disposal were differentially abundant in Jersey and Holstein rumen microbiomes

**Figure 4:**
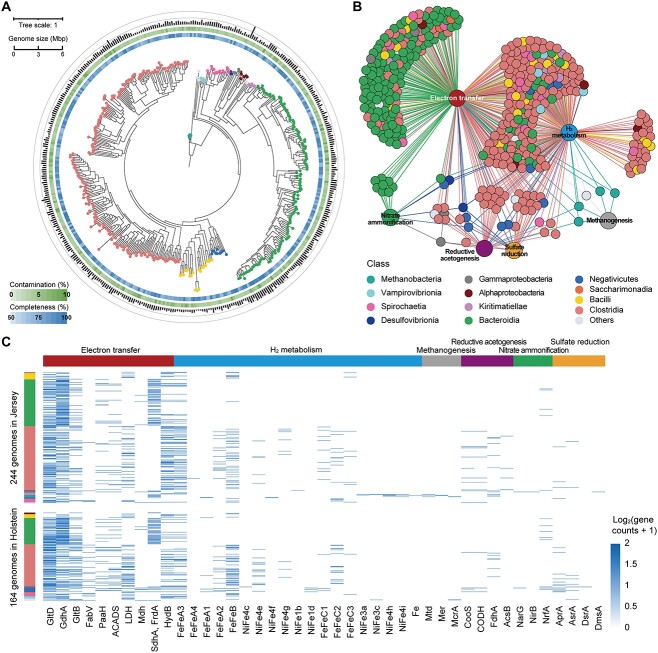
Phylogenetic tree and correlation network of 432 MAGs from Jersey and Holstein rumen microbiomes; (A) the maximum-likelihood tree with 432 MAGs; clades are colored according to the taxonomic classification of genomes; the heatmaps in the outer layer show the percentage of contamination and completeness corresponding to each genome, and the height of the outermost bar represents the genome size; (B) correlation network of metabolic pathways and genomes, with genomes colored according to taxonomic information; (C) heatmap for selected key metabolic genes in differentially enriched genomes in Jersey or Holstein rumen microbiome; key metabolic genes include reductant disposal via electron transfer during carbohydrate degradation and glutamate synthesis (red), molecular H_2_ metabolism (blue), methanogenesis (grey), reductive acetogenesis (purple), nitrate ammonification (green), and sulfate reduction (yellow).

To resolve the mediators of metabolic processes, we co-assembled and binned all metagenomes, yielding 432 high- or medium-quality metagenome-assembled genomes (MAGs) spanning 16 bacterial and 2 archaeal classes (Fig. 4A; Supplementary File S9). Consistent with the community composition of the rumen samples (Fig. 1F and Supplementary Figs S5 and S6), more than half of the genomes were affiliated with the dominant ruminal classes *Bacteroidia* and *Clostridia* [43, 44]. We then searched for key metabolic genes in the derived genomes (Fig. 4B; Supplementary File S9). As anticipated, a total of 376 MAGs (87%) showed the capability to dispose of reductant through electron transfer during glutamate, propionate, and butyrate synthesis and were distributed in all 18 classes (mainly *Bacteroidia* and *Clostridia*). A total of 222 MAGs (51.3%) showed the capability to produce or consume H_2_ via [FeFe]-hydrogenases (92%) and [NiFe]-hydrogenases (18%), whereas only one genome encoded [Fe]-hydrogenases (Fig. 4C). Although these hydrogenase-positive MAGs spanned 13 classes, most were affiliated with *Clostridia*. Of these 222 MAGs, 110 encoded enzymes for fermentative H_2_ production (*Clostridia* = 74.5%, *Bacilli* = 7.3%), whereas 10 MAGs encoded H_2_-uptake hydrogenases. In addition, we assembled multiple MAGs encoding key metabolic genes involved in methanogenesis (5 MAGs), reductive acetogenesis (29 MAGs), nitrate ammonification (14 MAGs), and sulfite reduction (22 MAGs) (Fig. 4B and C). These results indicate that most of the genomes assembled had the capacity to dispose of reductant through various pathways in the rumen microbial ecosystem.

We next investigated the relative abundance of the assembled genomes. One million random metagenomic reads from each sample were extracted and aligned to the genomes, resulting in an average mapping rate of 41% (Supplementary Table S6). We found that 244 and 164 MAGs encoded key metabolic genes were more enriched in Jersey and Holstein rumen samples, respectively (Fig. 4C; Supplementary File S10). Specifically, MAG60 (*Succiniclasticum* sp.) and MAG222 (*Succinivibrio* sp.), predicted to incorporate electrons during lactate and glutamate synthesis, were significantly enriched in the Holstein rumen microbiome (*P* < .05; 8.7- and 5.0-fold higher). MAG85 (RF16 group), which is predicted to catalyze the reduction of oxaloacetate to malate in propionate production, was enriched in the Jersey rumen microbiome (*P* < .01; 4.0-fold higher). This seems inconsistent with the lower propionate production in the Jersey rumen microbiome, but the physiology of the RF16 group of *Bacteroidia* has not been elucidated, and the role of this pathway in these bacteria remains to be understood. MAG344 (*Megasphaera elsdenii*), a potent fermenter of lactate [45] and predicted to be involved in butyrate production, was enriched in the Holstein microbiome (*P* < .01; 4.5-fold higher). Among the hydrogenase-positive genomes, MAG41 (CAG-603), which encodes fermentative hydrogenases, was more abundant in the Jersey rumen microbiome (*P* < .001; 7.6-fold higher). The MAG34 (RUG114 sp.), which encoded respiratory hydrogenases, was more abundant in the Holstein rumen microbiome (*P* < .001; 101-fold higher). Furthermore, four out of five genomes from putatively methanogenic microbes (i.e. MAG399, *Methanobrevibacter*, *P* < .001; 16.8-fold higher), which harbored unique hydrogenases (group 3a, 3c, 4 h, 4i [NiFe]-hydrogenases and [Fe]-hydrogenases) and the signature gene *mcrA*, were enriched in Jersey rumen microbiome. Moreover, *Selenomonas_*C *bovis*-affiliated MAG89, encoding both adenylylsulfate- and nitrate-reducing genes, was significantly enriched in the Holstein rumen microbiome (*P* < .001; 20.6-fold higher). Together, genomes of organisms capable of incorporating electrons for metabolite synthesis and using sulfate and nitrate as H_2_ acceptors were enriched in the Holstein rumen microbiome, and genomes of organisms capable of fermentative H_2_ production and hydrogenotrophic methanogenesis were enriched in the Jersey rumen microbiome.

We investigated whether these differences partially reflect selection for different microbiota growth rates, for example since rumen passage times are potentially faster in Jersey cattle due to their smaller size. To do so, we employed the Growth Rate InDex (GRiD) algorithm [46] to estimate the growth rates of the MAGs. Most MAGs had a low growth rate (GRiD <1.2). MAG25, affiliated with *Clostridia*, was the only bacterial group with a higher GRiD score being around 1.7 (Supplementary File S11). However, the growth rates of these species were not significantly different between Jersey and Holstein rumen (*P* > .05; Supplementary Fig. S14 and Supplementary File S11). These results indicate that the impact of different host breeds on microbial growth rates appears to be relatively minor. However, other breed-related differences in anatomy and physiology are likely to be a strong selective pressure resulting in differences in microbiota composition, function, and activities.

### *In vitro* incubations verify differences in microbiota functions

**Figure 5:**
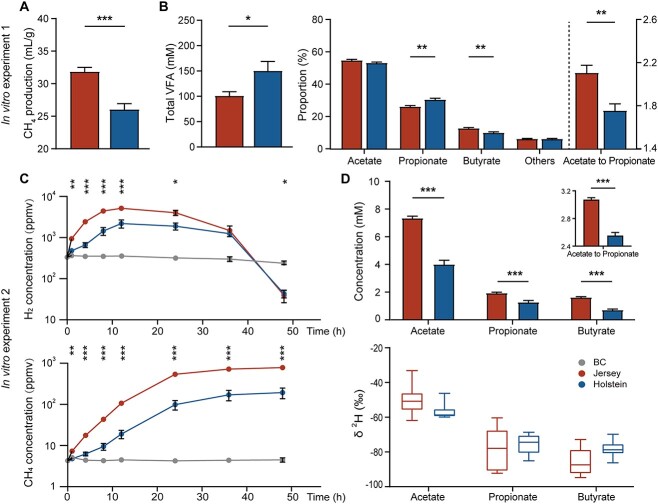
Metabolic activities of Jersey and Holstein rumen microbiomes; (A) CH_4_ production; (B) VFAs concentrations, and molar proportion of individual VFA in *In Vitro* Rumen Experiment 1 with feed fermented with Jersey or Holstein rumen inocula; (C) time course of gases hydrogen (gH_2_) and methane (gCH_4_) concentration in *In Vitro* Experiment 2; (D) VFA concentration, acetate to propionate, and fractionation of stable deuterium isotopes in 48-h acetate, propionate, and butyrate in *In Vitro* Rumen Experiment 2 inoculated with Jersey or Holstein rumen contents; BC, blank control; data with error bars are expressed as mean ± standard error; ^*^P < .05, ^*^^*^P < .01, ^*^^*^^*^P < .001, *n* = 6.

In Vitro Experiment 1 was carried out to compare the activity of Jersey and Holstein rumen microbiomes by incubating the same total mixed rations (TMRs) substrate with rumen inoculum from the selected dairy cows. The Jersey rumen microbiome produced more CH_4_ (*P* < .001; Fig. 5A1.3-fold higher), whereas the Holstein rumen microbiome produced greater *in vitro* VFA concentrations (*P* < .01; Fig. 5B; 1.5-fold higher), which agrees with *in vivo* findings of greater capacity for carbohydrate metabolism (Fig. 3A). Furthermore, the Holstein rumen microbiome fermented the TMR to a higher molar proportion of propionate, lower molar proportion of butyrate and acetate to propionate ratio (*P* < .05; Fig. 5B). The results agree with the *in vivo* analyses presented above, and verified again lower CH_4_ and more propionate production by the Holstein rumen microbiota, in agreement with the well-known inverse relationship between propionate and CH_4_ production [21].

To compare the H_2_ utilization capacities of Jersey and Holstein rumen microbiota, *In Vitro* Experiment 2 was performed by adding isotopically labeled H_2_ (molecular deuterium, D_2_, or ^2^H_2_). The headspace gH_2_ concentration rapidly increased during the initial hours of incubation, reaching a peak after 10 h, and finally decreased to a value below the uninoculated controls (Fig. 5C). Such increases in headspace gH_2_ concentration were likely caused by the fermentation of residual feed material in the rumen inoculum and are in agreement with the results of the incubation of TMR feed as a substrate (Fig. 1D). The amount of gH_2_ formed was much greater than that added as ^2^H_2_. The Jersey rumen inoculum resulted in a greater peak gH_2_ concentration (*P* < .001; Fig. 5C; 5144 ppmv vs. 2189 ppmv) and lower 48 h gH_2_ concentration (*P* < .05; Fig. 5C; 18.8 ppmv vs. 43.0 ppmv) than the Holstein rumen inoculum. Furthermore, CH_4_ concentrations were greater for incubations with Jersey rumen inocula (*P* < .01; Fig. 5C). These results indicated that methanogens in the Jersey rumen inocula used more H_2_ than Holstein rumen inocula. Additional H_2_ supplementation increases VFA production by favoring acetate production [47], which can be caused by enhanced reductive acetogenesis and its conversion to propionate and butyrate [48-50] or shifts in fermentation of the residual substrates [51]. Jersey rumen inocula showed a greater ability to incorporate headspace ^2^H_2_ for VFA production than Holstein rumen inocula. The increased acetate concentration with a greater acetate-to-propionate ratio (*P* < .05; Fig. 5D) was consistent with increased acetogenesis transcripts in the rumen of Jersey cows. However, no significant differences were observed in the abundance of ^2^H in individual VFAs (*P* > .05; Fig. 5D). Altogether, Jersey rumen microbiota showed a greater ability to utilize H_2_ for CH_4_ and homoacetogenic acetate production.

### Reductant disposal via electron transfer and H_2_ metabolism is widespread and correlated with CH_4_ yield

**Figure 6:**
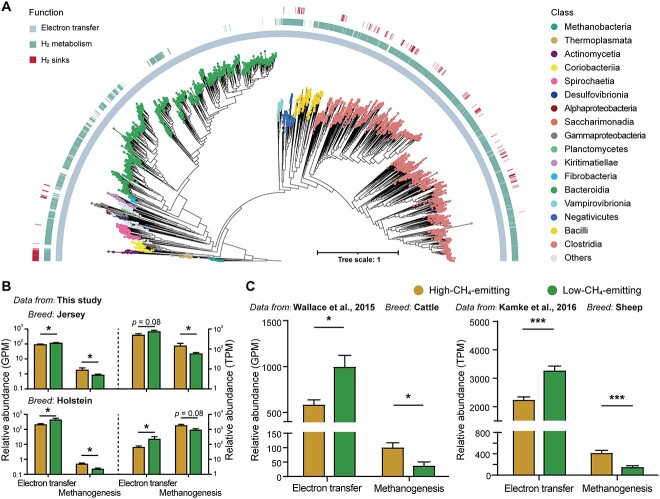
Diverse reductant disposal pathways via electron transfer or H_2_ metabolism in ruminants; (A) maximum-likelihood tree of the global public 7651 MAGs database derived from the superset developed by Stewart *et al*. [52] and binned genomes from Xie *et al*. [53], and their associations with reductant disposal via electron transfer during carbohydrate degradation and glutamate synthesis, molecular H_2_ metabolism and H_2_ sinks for methanogenesis, reductive acetogenesis, nitrate ammonification, and sulfate reduction. The heatmaps in the outer layer show whether the corresponding genome has functions (colored) or not (blank); clades are colored according to the class of genomes; (B) a comparison of the abundance of maker genes involved in reductant disposal via electron transfer during carbohydrate degradation and glutamate synthesis and H_2_ incorporation into methanogenesis in rumen metagenome and metatranscriptome between high-CH_4_-emitting (yellow) and low-CH_4_-emitting (green) Jersey or Holstein (*n* = 4) from this study; (C) a comparison of the abundance of maker genes involved in reductant disposal via electron transfer during carbohydrate degradation and glutamate synthesis and H_2_ incorporation into methanogenesis in rumen metagenome between high- and low-CH_4_-emitting beef cattle (*n* = 4) from Wallace *et al*. [31] and metatranscriptome of high-CH_4_-emitting and low-CH_4_-emitting sheep (*n* = 8) from Kamke *et al*. [54]; data with error bars are expressed as mean ± standard error; ^*^P < .05, ^*^^*^^*^P < .001.

We gained a global perspective of the distribution of genes involved in reductant disposal reactions and H_2_ metabolism by searching the 7651 global rumen metagenomes generated by Stewart *et al*. [52] and Xie *et al*. [53]. Similar to our binning results, 94.6% of these genomes encoded for genes involved in reductant disposal (Fig. 6A; Supplementary File S12). Most genomes that could synthesize glutamate and produce propionate were associated with *Clostridia* and *Bacteroidia*. A total of 61.1% of genomes encoded hydrogenases including [FeFe]-hydrogenases (92.8%), [NiFe]-hydrogenases (20.2%), and [Fe]-hydrogenases (1.5%), which were mainly affiliated with *Clostridia*. Relatively few genomes (4.9%) were identified as hydrogenotrophs, including methanogens belonging to *Methanobacteria* (which includes *Methanobrevibacter* and *Methanosphaera*) and *Thermoplasmata* (which includes *Methanomassiliicoccales*), reductive acetogens (*Clostridia*), and respiratory hydrogenotrophs (*Desulfovibrionia*).

To verify the universality of our results obtained by comparing the Jersey and Holstein rumen microbiomes, we further selected four high- and four low-CH_4_-emitting Holstein or Jersey cows within each group according to the CH_4_ yield (Supplementary Fig. S15). Both the metagenomic and metatranscriptomic analyses indicated that low-CH_4_ emitters had greater gene abundances involved in electron transfer and lower gene abundances involved in methanogenesis (Fig. 6B). We also reanalyzed the relative abundance of the key metabolic genes in the rumen metagenome and metatranscriptome of high-CH_4_-emitting and low-CH_4_-emitting sheep or beef cattle in public datasets obtained from New Zealand and the UK [31, 54]. Consistent with our results, the low-CH_4_-emitting sheep and cattle had a greater relative abundance of genes involved in reductant disposal via electron transfer for organic fermentation product synthesis, whereas high-CH_4_ emitters had a higher abundance of genes involved in H_2_ utilization via methanogenesis (*P* < .05; Fig. 6C). These results again support that the two CH_4_-emitting phenotypes exhibit quantitative variations in the flows of pathways of reductant disposal, with greater ability to incorporate electron for VFA production and glutamate synthesis in low-CH_4_ emitters and incorporate H_2_ for methanogenesis in high-CH_4_ emitters.

## Discussion

**Figure 7:**
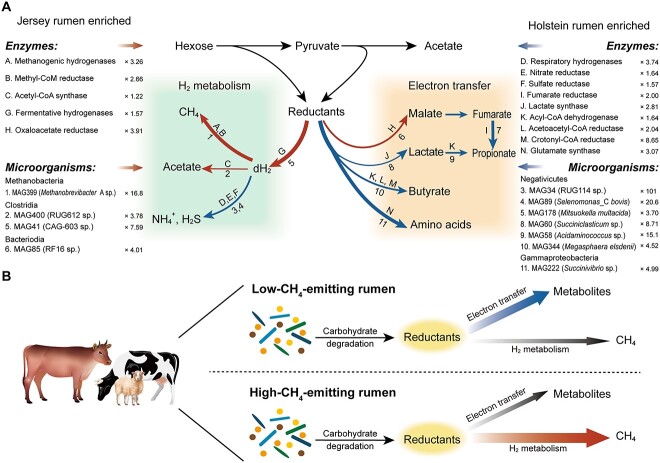
Rumen microbiomes exhibit distinct pathways and capacities of reductant disposal via electron transfer or H_2_ metabolism; (A) Jersey rumen (red): less reductant was disposed of via electron transfer to produce host-beneficial metabolites, while more reductant was disposed of via H_2_ production, which was consumed by methanogens to produce CH_4_ and by reductive acetogens to produce acetate; Holstein rumen (blue): more reductant was disposed of via electron transfer through producing host-beneficial metabolites, and less reductant was disposed of via H_2_ production, which was relatively more favorable for nonmethanogenesis pathways, such as nitrate and sulfate reduction; the key enzymes, representative MAGs, and the fold differences are shown for (left) Jersey/Holstein and (right): Holstein/Jersey; these data are presented in detail in Supplementary File S13; red lines, enhanced in Jersey rumen, and blue lines, enhanced in Holstein rumen; the arrow thickness shows the relative importance based on metatranscriptomic data; (B) proposed rumen H_2_ metabolism models; in the low-CH_4_-emitting rumen, reductant was disposed of via electron transfer to host-beneficial metabolites (bold blue line), whereas in the high-CH_4_-emitting rumen, reductant was disposed of via H_2_ metabolism to produce CH_4_ (bold red line).

The Holstein rumen microbiota had a greater proportion of reductant disposal via electron transfer for amino acid synthesis and reduced VFA production catalyzed by enzymes such as glutamate synthase and lactate dehydrogenase bypassing H_2_ production and thus leading to the less expelled H_2_ and CH_4_ (Fig. 7A). The lower CH_4_ yield of Holsteins did not translate into greater DMI-adjusted milk production, which is in agreement with meta-analyses in which inhibiting CH_4_ formation in the rumen did not result in consistent benefits in animal productivity [55, 56]. The Jersey rumen microbiome had a greater proportion of reductant disposal via H_2_ production catalyzed by fermentative hydrogenases encoded by *Clostridia*, with H_2_ mainly taken up by *Methanobrevibacter* through enriching methanogenic hydrogenases (Fig. 7A). Genetic and physiological differences do exist between the two breeds, which could potentially influence microbial dynamics and metabolism, leading to different CH_4_ emission phenotypes. Our breed-specific analyses, along with *in vitro* experiments and public data validation, support the mechanism of enhanced electron incorporation for metabolite synthesis and reduced methanogenesis in low-CH_4_-emitting sheep and cattle.

The H_2_ production and utilization processes are tightly coupled during the fermentation of ingested feed to maintain a low H_2_ concentration so that the fermentation can continuously proceed. The host phenotype of CH_4_ emissions is the result of diverse fermentative H_2_ production and incorporation pathways [24]. The variation in the coupling of fermentative H_2_ production and incorporation can be associated with different VFA production and microbial cell synthesis profiles, which exhibit differential expression of pathways in both reductant disposal and H_2_ metabolism. Propionate and MCP synthesis are related to net reductant disposal [16, 21], and propionate and MCP production increases are generally associated with reduced hydrogenogenesis and CH_4_ production [16, 57, 58]. Such propionate and microbial cell synthesis enhancement is consistent with decreased enrichment of fermentative hydrogenases and lower dH_2_ concentrations in the Holstein rumen, leading to an enhancement in reductant disposal via electron transfer and reduction in hydrogenogenic processes. Reanalysis of the other rumen metagenome and metatranscriptome data indicates that the enrichment of rumen microbiome enrichment genes of electron transfer for metabolite synthesis may be universal in naturally low-CH_4_ emitters.

Methanogenesis is the major H_2_ sink in the rumen microbial ecosystem and is assumed to help maintain a low steady-state H_2_ concentration in the rumen. The importance of methanogenesis as the main H_2_ sink is reflected by the higher expression level of *mcrA* compared with other reductases [30]. In Jersey cattle, CH_4_ concentration and production was 1.2-fold higher than Holstein counterparts, concomitant with an increased abundance of the methanogen *Methanobrevibacter* and genes for the methane-producing enzyme Mcr, indicating higher rates of hydrogenotrophic methanogenesis. However, methylotrophic methanogens (i.e. *Methanosphaera*, Ca. *Methanomethylophilus*) were more abundant in Holstein cattle over hydrogenotrophic methanogens (i.e. *Methanobrevibacter*), suggesting the methylotrophic pathway accounts for a greater proportion of CH_4_ emissions in this breed. Methylotrophic methanogens have an energetic advantage over hydrogenotrophic methanogens at the lower hydrogen partial pressures found in their rumen [59, 60]. This may offer an explanation for the observed substantial reduction of *Methanobrevibacter* with a more moderate reduction in CH_4_ emissions in Holstein cattle. A reduction of the abundance of *Methanobrevibacter* spp. relative to methylotrophic methanogens was also observed in a study of naturally low-CH_4_ emitting sheep and was attributed to decreased flux of H_2_ to hydrogenotrophic methanogens [61].

Multiple alternative hydrogenotrophic pathways operate in the rumen and compete with methanogens for ruminal H_2_ supply. The Jersey rumen microbiome exhibited an enhancement of hydrogenotrophic acetogenesis assigned to *Clostridia*. The higher concentration of dH_2_ in the Jersey rumen might satisfy the H_2_ threshold concentrations required for reductive acetogens so that reductive acetogenesis could be thermodynamically favored [25]. Holstein rumen microbiome exhibited a potential enhancement for nitrate and sulfate reduction by *Bacteroidia* and *Negativicutes*, which is in agreement with the reports that alternative H_2_ uptake pathways such as nitrate and sulfate respiration might collectively serve as alternative H_2_ sinks to methanogenesis in low-CH_4_-emitting sheep [24]. Nitrate and sulfate reducers have lower H_2_ thresholds than other hydrogenotrophs and so compete effectively for H_2_ at low concentrations if not limited by the availability of their electron acceptor [25]. Altogether, we demonstrate that the host phenotype of CH_4_ emissions exhibits distinct pathways of H_2_ utilization through nonmethanogenesis pathways.

Overall, the host phenotype of CH_4_ emissions exhibits distinct microbial H_2_ and reductant disposal pathways (Fig. 7B). Low-CH_4_-emitting rumen shows an enhanced ability to dispose of reductant via electron transfer for host-beneficial metabolite synthesis, whereas high-CH_4_-emitting rumen exhibits a larger proportion of reductant disposal via H_2_ production and its use by the enriched methanogenic H_2_-consuming pathway. These findings provide new insights into the basis of variation of CH_4_ emissions in ruminants and underlying ecological mechanisms that control H_2_ transactions in microbial communities. As prokaryote associated with feed particles is undersampled by the stomach tubing methodology that can potentially influence the actual differences in protein amounts and the relative abundance of individual taxa, further works are needed to investigate the underlying host-specific mechanisms that select for different microbiome structures and activities in both liquid and solid phase of bovine rumen. These specific differentially enriched genes, metabolites, and microorganisms could serve as biomarkers to potentially aid breeding programs and dietary formulations to lower CH_4_ emissions from ruminants.

## Materials and methods

Full details of the abbreviated methods below, together with microbial DNA and RNA extractions, qPCR, biodiversity analysis, and isotope ratio measurement, are included in the Supplementary Materials and Methods.

### Experimental setup and sampling

The experiment was conducted at Youzhuo Animal Husbandry Center, Hunan Province, China (28.408871 N, 112.669765 E) and approved by the Animal Care Committee (approval number: W201902) of the Institute of Subtropical Agriculture, the Chinese Academy of Sciences.

The experiment included 12 second parity Jersey (initial body mass = 507 ± 20.8 kg, mean ± SEM) and 12 second parity Holstein (initial body mass = 636 ± 12.9 kg, mean ± SEM) dairy cows, which were selected to represent each of their herds at the dairy farm based on their milk yields of the first parity. Experimental cows had no previous record of diseases, miscarriages, or twin pregnancies. The first parity milk yields of the selected animals were normally distributed within each breed (Jersey: 6670 ± 270 kg/parity, Holstein: 8250 ± 270 kg/parity, Fig. 1A). A total-mixed ration (TMR) with 51% of concentrate (Supplementary Table S1) was offered *ad libitum* to all cows. Water was always available for all animals. Measurements and sampling began at 220 days in milk and comprised 5 days for measuring milk production and nutrient digestibility, 2 days for sampling rumen content, and 2 days for measuring enteric CH_4_ production.

### Animal measurements

Cows were milked three times daily (06:00, 14:00, and 22:00), and milk production was recorded (DeLaval, Sweden). Milk samples were collected for the estimation of milk composition (i.e. fat, protein, and lactose) using a spectrophotometer through infrared analysis (MilkoScanTM FT; Foss Electric) [62]. FCM was calculated using the equation of Gaines [63]. Enteric CH_4_ emissions were measured in respiration chambers [64].

### Rumen sample collection and analysis

Feed intake for each cow was recorded daily. Fecal samples were collected from the rectum for approximate composition analysis [65], and acid-insoluble ash was used as an endogenous marker to calculate apparent nutrient digestibility [66]. Rumen samples were collected at 0 and 2.5 h after morning feeding using a stomach tube, with the first 150 ml of rumen contents discarded to avoid saliva contamination [22]. Ruminal pH, dissolved hydrogen (dH_2_), and dCH_4_ were first measured from the liquid phase of rumen contents as previously described [22]. Ammonia concentrations were determined by the method of Weatherburn [67]. Microbial cells in rumen fluid were separated by differential centrifugation according to a published method [68], and the MCP concentration in rumen fluid was measured colorimetrically according to the Bradford protein assay [69]. Individual VFAs concentrations were analyzed by gas chromatography as described by a previous study [70]. Targeted metabolomics of central carbon metabolism in rumen liquid samples was conducted by positive/negative Multiple Reaction Monitoring at Novogene Co., Ltd (Beijing, China).

### 16S ribosomal RNA gene sequencing

The composition and diversity of the bacterial and archaeal communities in Jersey and Holstein rumen microbiomes were analyzed through 16S rRNA gene sequences. For bacteria, the V3 and V4 regions of the 16S rRNA gene were amplified with a barcode by using the universal primers (341F: 5′-CCTAYGGGRBGCASCAG-3′, 806R: 5′-GGACTACNNGGGTATCTAAT-3′) [71]. For methanogenic archaea, a specific primer set was selected (86F: 5′-GCTCAGTAACACGTGG-3′, 471R: 5′-GWRTTACCGCGGCKGCTG-3′) [72]. Amplicon sequencing and analysis of rumen prokaryotic composition were performed as described by a previous study [73], with all amplicon libraries being sequenced on the MiSeq platform (Illumina, San Diego, CA) at Shanghai Biozeron Biological Technology Co. Ltd. After removing barcodes and sequencing primers, passed sequences were dereplicated and subjected to the DADA2 algorithm to identify indel-mutations and substitutions [74], to generate ASVs. The phylogenetic affiliation of each 16S rRNA gene sequence was analyzed by RDP Classifier (http://rdp.cme.msu.edu/) against the SILVA (SSU138) database using a confidence threshold of 70%.

### Metagenome sequencing, assembly, and binning

The specific software, parameters, and databases used in the present study are given in the Supplementary Materials and Methods. Briefly, sequencing was performed on the HiSeq X platform (Illumina, San Diego, CA) with pair-end 150 bp (PE150) mode at Shanghai Biozeron Biological Technology Co. Ltd. Low-quality reads, contaminated adaptors, and host reads (Bos_taurus. UMD3.1 [75] and hg19 [76]) were removed. An average of 13.5 gigabases of paired-end reads per sample was obtained for further analysis. MEGAHIT [77] was used to predict the contigs from each sample, and Prodigal [78] was used to predict the contigs. Subsequently, the ORFs derived from assembled contigs were maintained and clustered into a nonredundant dataset by CD-HIT [79], which was further used to analyze the subsequent predicted metagenomic function of the rumen microbiome. The abundance profile of genes was calculated and transformed to gene per million (GPM) [80], with corrections for variations in gene length and mapped reads per sample.

Metagenomic binning was performed for each sample to obtain MAGs. We used metaBAT2 [81] to perform separate binning. All bins with completeness >50% and contamination <10% were considered “filtered bins” after verifying with CheckM [82]. All MAGs were dereplicated with a 99% ANI cutoff using dRep [83] to obtain 432 nonredundant MAGs. The taxonomy of each genome was annotated by GTDB-Tk [84] based on the Genome Taxonomy Database, and the relative abundance of each MAG in each sample was calculated according to the GPM calculation process by aligning high-quality reads BWA-MEM [84]. The GRiD algorithm (v.1.3) was used to estimate the growth rates of each MAG by calculating the coverage in replication origin and terminal origin [46]. Gephi [85] was used to construct the correlation network based on the key genes involved in pathways of H_2_ metabolism; if a MAG encodes the metabolic genes, there is a connecting line between the metabolic process and it. Integrated public MAGs were created using the superset database built by Stewart *et al*. [52] and the rumen MAGs published by Xie *et al*. [53]. Following de-replication with a 99% ANI cutoff using dRep, 7651 public rumen MAGs were obtained for further analysis. The taxonomy of clean metagenomic reads from each sample was generated with GraftM software [86]. The proportion of community members that encode each gene was estimated by dividing the read counts for the gene (in reads per kilobase million [RPKM]) by the mean of the read counts of 14 universal single-copy ribosomal marker genes (in RPKM).

### Metatranscriptome sequencing and mapping

Metatranscriptome sequencing was performed using a HiSeq 2500 System (Illumina). Paired-end reads were generated with 150 bp in the forward and reverse directions. After removing host DNA and adaptor contaminants, a set of high-quality reads with an average of 6.1 gigabases paired-end reads for each sample was obtained for further analysis. Relative gene expression values were calculated as described elsewhere [87]. Generally, each sample’s metatranscriptomic reads were mapped to predicted ORFs obtained in metagenomes, and TPM values were employed in this study.

### Functional annotation and phylogenetic analysis

To compare the metabolic capability of rumen microbial communities, the metagenomes, metatranscriptomes, and MAGs were searched against a local protein database of representative metabolic genes related to reductant disposal reactions involved in carbohydrate degradation and amino acids synthesis, molecular H_2_ production and incorporation, and marker genes for key metabolic processes. Searches were carried out through hidden Markov models (HMMs) and homology-based searches. Genes were also annotated against the KEGG database for analyzing carbohydrate metabolism by HMM searches with default parameters and then summarized through the abundance of Level 3 and Level 2 pathways, along with KOs involved in VFA production [73].

Key enzymes of reductant disposal via electron transfer reactions involved in carbohydrate degradation and amino acid synthesis included glutamate synthase (GdhA, GltB, GltD), propionate production (SdhA, FrdA, Ldh, Mdh, ACADS), and butyrate production (PaaH, FabV, Supplementary File S4). These enzymes were annotated by HMM searches against a local protein database, except that the sdhA and frdA were annotated by BLAST against a custom database [24]. Hydrogenases related to H_2_ production and incorporation (i.e. NiFe-, FeFe-, and Fe-hydrogenases) were identified with the HydDB database by DIAMOND [88] (v.2.0.4) with an e-value threshold of 1e-50, one maximum target sequence per query, and results were then filtered (length of amino acid >40 residues, sequence identity >60%). Marker genes for key metabolic processes were searched for genes involved in methanogenesis (*mcrA*, *mer*, *mtd*), reductive acetogenesis through the Wood–Ljungdahl pathway (*acsB*, *coos*, *codH*, *fdhA*), nitrate ammonification (*narA*, *narG*, *nosZ*, *norB*, *nirB*, *nrfA*, *nirK*, *nirS*), and sulfate reduction (*aprA*, *asrA*, *dsrA*, *asrB*, *dsmA*). Marker genes of *acsB*, *aprA*, *asrA*, *cooS*, *dmsA*, *dsrA*, *mcrA*, *napA*, *narG*, and *nrfA* were identified by DIAMOND searches against respective custom gene sequences referring to a previous publication [24], whereas other marker genes were obtained by HMM searches against a local protein database. Detailed information is listed in Supplementary File S4. Genes were subjected to the NCBI-NR (October 2018; ~550 M sequences) for a taxonomic and functional assignment using DIAMOND [88] (v.2.0.4) based on BLASTP searches. The gene set was also aligned with the CAZy database [89] using HMMER [90] (v.3.3.1) to harvest corresponding annotations for CAZyme. The phylogenetic tree of functional protein sequences and MAGs was constructed by FastTree [91] and PhyloPhlAn [92], respectively, and then visualized by iTOL [93].

### Measurements of microbiome activity through *in vitro* experiments

Detailed information is provided in the Supplementary Materials and Methods. The first *in vitro* experiment (“*In vitro* Experiment 1”) was conducted to compare the fermentative activities, including gases and VFA production of Jersey and Holstein rumen microbiomes by incubating the TMR fed to the animals according to a previously published procedure [94]. *In vitro* batch cultures were incubated at 39.5°C for 48 h. CH_4_ and H_2_ concentrations were determined through gas chromatography (Agilent 7890A, Agilent Inc., Palo Alto, CA) [22]. Methane production was then calculated by using the equation of Wang *et al*. [95]. The second *in vitro* experiment (“*In vitro* Experiment 2”) was conducted to compare the activities of Jersey and Holstein rumen microbiomes to use H_2_ by adding molecular deuterium (^2^H_2_ or D_2_) to the incubation bottle headspace. Samples of each bottle headspace (2 ml) were collected at 1, 4, 8, 12, 24, 36, and 48 h of incubation and used to measure headspace H_2_ and CH_4_ concentrations through gas chromatography (Agilent 7890A, Agilent Inc., Palo Alto, CA) [22]. Samples from the liquid phase of the bottle in the two experiments were collected from each bottle after finishing the incubations and snap frozen in liquid N_2_, and stored at −80°C for further analysis of fermentation end products.

### Statistical analyses

The generalized linear model procedure was used to analyze the metabolites concentration and production using the SPSS 21.0 software (SPSS Inc., Chicago, IL). When sampling time was included in the model, a linear mixed model was used with treatment, sampling time, and the treatment by sampling time interaction as fixed effects and the animal as a random factor. The ANCOM-BC R package was used to determine the differentially abundant taxa for amplicon and metagenome data [96]. The Wilcoxon rank-sum test in the JMP Pro software (JMP Pro version 13.2.1, SAS Institute Inc., SAS Institute, Cary, NC) was used to analyze the relative abundance of functional genes and MAGs. All *P*-values were adjusted for false discovery rate using the Benjamini–Hochberg method, and *P* < .05 was regarded as statistically significant.

## Supplementary Material

1_Bacterial_16S_rRNA_gene_amplicons_information_wrad016

2_Archaeal_16S_rRNA_gene_amplicons_information_wrad016

3_Rawdata_of_central_carbon_metabolism_wrad016

4_Metadata_sequencing_statistics_and_community_of_metabolic_marker_genes_wrad016

5_KEGG_analysis_at_level_2_and_3_wrad016

6_CAZyme_statstics_and_nr_profiles_wrad016

7_NR_annotation_of_VFA_production_pathways_and_metabolic_genes_wrad016

8_Relative_abundance_of_metabolic_marker_genes_in_metagenome_and_metatranscriptome_wrad016

9_Quality_statistics_and_taxonomy_and_gene_information_of_the_432_wrad016

10_Relative_abundance_of_432_MAGs_wrad016

11_GRiD_values_of_MAGs_in_each_sample_wrad016

12_Taxonomy_and_gene_information_of_the_7651_public_metagenome-assembled_genomes_wrad016

13_Consolidation_of_results_from_enzymes_and_genomes_recruitment_analysis_wrad016

Supplementary_materials_wrad016

## Data Availability

Amplicon, metagenomic, and metatranscriptomic sequences are available at the National Center for Biotechnology Information (NCBI, project number PRJNA868624, PRJNA869873, and PRJNA870639, respectively). All other data supporting the results of this study are available in the article or Supplementary information.
