# Supplementary material for: Distinct microbial hydrogen and reductant disposal pathways explain interbreed variations in ruminant methane yield
Source: ISME J. 2024 Jan 10;18(1):wrad016. doi: 10.1093/ismejo/wrad016 (PMC10811737; doi:10.1093/ismejo/wrad016)
Supplement: Supplementary_materials_wrad016 [file supplementary_materials_wrad016.docx]

**Supplementary information for**

**Distinct microbial hydrogen and reductant disposal pathways explain** **interbreed variations in ruminant methane yield**

Qiushuang Li ^1, 2, 8^, Zhiyuan Ma ^3, 8^, Jiabin Huo ^1^, Xiumin Zhang ^1^, Rong Wang ^1^, Shizhe Zhang ^1^, Jinzhen Jiao ^1^, Xiyang Dong ^4^, Peter H. Janssen ^5^, Emilio M. Ungerfeld ^6^, Chris Greening ^7^, Zhiliang Tan ^1,2^, Min Wang ^1, 2*^

^1^ *Key Laboratory for Agro-Ecological Processes in Subtropical Region, Institute of Subtropical Agriculture,* *Chinese Academy of Sciences, Changsha, Hunan, China*

^2^ *University of Chinese Academy of Sciences, Beijing, China*

^3^ *College of Pastoral Agriculture Science and Technology, Lanzhou University, Lanzhou, China*

^4^ *Key Laboratory of Marine Genetic Resources, Third Institute of Oceanography, Ministry of Natural Resources, Xiamen, China*

^5^ *AgResearch Limited, Grasslands Research Centre, Palmerston North, New Zealand.*

^6^ *Centro Regional de Investigación Carillanca, Instituto de Investigaciones Agropecuarias (INIA), Temuco, Chile*

^7^ *Biomedicine Discovery Institute, Department of Microbiology, Monash University, Clayton, Australia*

^8^ These authors contributed equally to this work

^*^E-mail: mwang@isa.ac.cn (M.W.)

Competing interests: All the authors declare they have no competing interests.

This PDF file includes:

Additional Table S1 to S7

Additional Figures S1 to S15

Supplementary Materials and Methods

**Additional Tables**

**Table S1.** Ingredients and chemical compositions of diet.

**Table S2.** Information on selected Jersey and Holstein cows.

**Table S3.** Comparison of feed intake, milk production, and apparent digestibility between Jersey and Holstein dairy cows.

**Table S4.** Comparison of methane emission between Jersey and Holstein dairy cows.

**Table S5.** Comparison of rumen fermentation parameters between Jersey and Holstein dairy cows. **Table S6.** 432 MAG**s** mapping rate of 24 metagenomic samples.

**Table S7**. Primer sequences for q-PCR of target species.

**Additional Figures**

**Figure S1.** Bacterial and archaeal rarefaction curves based on 16S rRNA gene sequence variants (ASVs).

**Figure S2.** Comparison of 16S rRNA copy numbers of bacteria, methanogens, and genus *Methanobrevibacter* between Jersey and Holstein rumens.

**Figure S3.** Comparison of alpha (ACE and Shannon index) of bacterial (A) or archaeal community (B) between Jersey and Holstein rumen microbiomes based on 16S rRNA gene amplicon sequence variants (ASV).

**Figure S4.** Comparison of relative abundance of bacterial phyla and genera between Jersey and Holstein rumen microbiomes based on 16S rRNA gene amplicon sequence variants (ASV) using ANCOM-BC analysis.

**Figure S5.** The distinct bacterial and archaeal community between Jersey and Holstein rumen based on metagenome.

**Figure S6.** The distinct bacterial and archaeal community between Jersey and Holstein rumen based on metatranscriptome

**Figure S7.** Comparison of Kyoto Encyclopedia of Genes and Genomes (KEGG) profile between Jersey and Holstein rumen metagenome.

**Figure S8.** Comparison of CAZymes profile between Jersey and Holstein-rumen metagenome.

**Figure S9.** Maximum-likelihood phylogenetic tree of amino acid sequences of FeFe hydrogenases.

**Figure S10.** Maximum-likelihood phylogenetic tree of amino acid sequences of NiFe hydrogenases.

**Figure S11.** Maximum-likelihood phylogenetic tree of amino acid sequences of Fe hydrogenases.

**Figure S12.** Phylogenetic distribution of *mcrA* genes in Jersey and Holstein cows at genus level based on metagenomes.

**Figure S13.** Comparison of metagenomic relative abundance and metatranscriptomic transcript of methyl-transferase key enzymes.

**Figure S14.** Comparison of Growth Rate InDex (GRiD) of MAGs between Jersey and Holstein-rumen metagenome.

**Figure S15.** The intensity of CH_4_ emissions correct for dry matter intake (solid dots) and fat-corrected milk production (hollow dots) from 12 Jersey cows (A) and 12 Holstein cows (B) used in the current study.

**Additional Files (Excel files)**

**File S1.** Bacterial 16S rRNA gene amplicons information.

**File S2.** Archaeal 16S rRNA gene amplicons information.

**File S3.** Rawdata of central carbon metabolism.

**File S4.** Metadata, sequencing statistics, and community of metabolic marker genes in metagenomic short reads.

**File S5**. KEGG analysis at levels 2 and 3.

**File S6.** CAZyme statstics and nr profiles.

**File S7.** NR annotation of VFA production pathways and metabolic genes.

**File S8.** Relative abundance of metabolic marker genes in metagenome and metatranscriptome.

**File S9**. Quality statistics and taxonomy and gene information of the 432 metagenome-assembled genomes from the metagenomes.

**File S10.** Relative abundance of 432 MAGs.

**File S11.** GRiD values of MAGs in each sample.

**File S12**. Taxonomy and gene information of the 7651 public metagenome-assembled genomes.

**File S13**. Consolidation of results from recruitment analysis of enzymes and genomes.

**Additional Tables**

**Table S1.** Ingredients and chemical compositions of diet

| Item | Diet |
| --- | --- |
| Ingredient composition (g/kg DM) | |
| Corn silage | 490 |
| Brewer's grains | 122 |
| Corn | 90.0 |
| Alfalfa | 61.0 |
| Soybean meal | 40.0 |
| Gross cottonseed | 37.0 |
| Sliced corn | 28.0 |
| Beet pulp | 24.0 |
| Cottonseed Meal | 22.0 |
| DDGS | 22.0 |
| Oats | 20.0 |
| Molasses | 20.0 |
| Sodium Bicarbonate | 2.2 |
| Fat powder | 2.0 |
| Extruded soybean | 1.6 |
| Magnesium oxide | 1.1 |
| Lysine | 1.8 |
| Methionine | 0.7 |
| Premix^1^ | 14.6 |
| Chemical composition, g/kg dry matter | |
| Organic matter | 923 |
| Starch | 237 |
| Neutral detergent fiber | 326 |
| Acid detergent fiber | 180 |
| Crude protein | 165 |

#### ^1^ The premix was formulated to provide (per kg of premix DM): NaHCO_3_ 9 mg, oil 8 mg, MgO 5 mg, [Lysine](javascript:;) 0.75 mg, methionine 0.28 mg, vitamin D 30,000 IU, vitamin E 780,000 IU, Fe 520 mg, Cu 430 mg, Mn 490 mg, Zn 2,020 mg, Co 6mg, I 26 mg, Se 14 mg, Ca 140 g, NaCl 120 g, P 20 g.

† Mean values from the analysis of three samples.

**Table S2.** Information on selected Jersey and Holstein cows

| **Cow ID** | **Breed** | **Body Weight, kg** | **Milk Production, kg/d** | **Fat Corrected Milk, kg/d** | **Dry Matter Feed Intake, kg/d** | **Calving Date** |
| --- | --- | --- | --- | --- | --- | --- |
| 45421 | Jersey | 661 | 27.4 | 31.4 | 17.5 | 2018/12/2 |
| 45434 | Jersey | 462 | 23.8 | 24.9 | 17.5 | 2018/12/6 |
| 45472 | Jersey | 457 | 26.2 | 27.0 | 18.3 | 2018/11/8 |
| 47351 | Jersey | 397 | 23.1 | 23.5 | 14.6 | 2018/12/7 |
| 47770 | Jersey | 485 | 26.2 | 29.1 | 18.1 | 2018/12/2 |
| 48166 | Jersey | 540 | 26.2 | 28.67 | 17.0 | 2018/11/24 |
| 48334 | Jersey | 574 | 23.7 | 25.3 | 15.6 | 2018/12/20 |
| 48655 | Jersey | 486 | 23.9 | 22.9 | 13.6 | 2018/12/27 |
| 48839 | Jersey | 494 | 25.3 | 27.0 | 16.8 | 2018/12/8 |
| 49285 | Jersey | 437 | 26.2 | 27.7 | 15.4 | 2019/1/8 |
| 49652 | Jersey | 577 | 22.5 | 18.9 | 14.2 | 2019/6/1 |
| 49670 | Jersey | 515 | 30.8 | 32.1 | 17.8 | 2018/11/19 |
| 17365 | Holstein | 570 | 36.4 | 37.0 | 20.6 | 2018/12/26 |
| 17270 | Holstein | 632 | 36.0 | 35.2 | 20.8 | 2019/1/2 |
| 31447 | Holstein | 676 | 34.8 | 35.9 | 21.6 | 2018/12/17 |
| 30889 | Holstein | 581 | 37.1 | 34.8 | 22.2 | 2018/12/11 |
| 19334 | Holstein | 616 | 37.6 | 38.9 | 21.5 | 2019/1/21 |
| 30662 | Holstein | 664 | 39.4 | 39.2 | 24.7 | 2018/11/11 |
| 29292 | Holstein | 576 | 33.8 | 33.3 | 20.3 | 2019/1/5 |
| 31260 | Holstein | 650 | 29.3 | 30.6 | 21.6 | 2018/11/19 |
| 19707 | Holstein | 665 | 38.8 | 37.7 | 25.2 | 2018/12/12 |
| 19627 | Holstein | 625 | 36.4 | 35.1 | 24.9 | 2018/12/11 |
| 18227 | Holstein | 682 | 36.2 | 32.3 | 22.0 | 2018/12/21 |
| 31408 | Holstein | 706 | 28.9 | 28.5 | 18.8 | 2018/12/6 |

| **Table S3.** Comparison of feed intake, milk production, and apparent digestibility between Jersey and Holstein dairy cows | | | | |
| --- | --- | --- | --- | --- |
| Item | Breed | | SEM | *P*-value |
|  | Jersey | Holstein |  |  |
| Body mass, kg | 507 | 637 | 18.1 | <0.001 |
| Dry matter intake |  |  |  |  |
| kg/d | 16.4 | 22.0 | 0.69 | <0.001 |
| g/g body mass | 32.8 | 34.7 | 0.906 | 0.29 |
| Milk components, % |  |  |  |  |
| Fat | 4.27 | 3.91 | 0.093 | 0.05 |
| Protein | 3.78 | 3.42 | 0.051 | <0.001 |
| Milk production, kg/d | | | | |
| Fat corrected milk^1^ | 26.5 | 34.9 | 1.11 | <0.001 |
| Milk fat yield | 1.09 | 1.38 | 0.046 | <0.001 |
| Milk protein yield | 0.96 | 1.21 | 0.163 | <0.001 |
| Dry matter intake-corrected milk production, kg/kg | 1.62 | 1.59 | 0.029 | 0.57 |
| Apparent digestibility, g/kg | | | | |
| Dry matter | 614 | 632 | 12.8 | 0.51 |
| Neutral detergent fiber | 471 | 511 | 16.2 | 0.23 |
| Acid detergent fiber | 347 | 409 | 21.6 | 0.16 |
| Crude protein | 614 | 635 | 17.9 | 0.56 |

^1^: Daily milk production in 305 d was corrected according to the milk fat component. The calculation formula is daily milk production×0.4+daily milk fat production×15.

| **Table S4.** Comparison of methane emission between Jersey and Holstein dairy cows | | | | |
| --- | --- | --- | --- | --- |
| Item | Breed | | SEM | *P*-value |
|  | Jersey | Holstein |  |  |
| Daily methane emission |  |  |  |  |
| g | 245 | 313 | 17.2 | 0.044 |
| mg/kg Body mass | 476 | 492 | 30.5 | 0.81 |
| g/kg DMI^1^ | 30.3 | 19.7 | 2.68 | 0.047 |
| g/kg FCM^2^ | 12.2 | 9.1 | 0.522 | 0.008 |
| Energy in methane/Energy intake, J/100J | 8.64 | 5.64 | 0.698 | 0.029 |
| Energy in methane/Energy digested, J/100J | 14.0 | 9.21 | 1.20 | 0.042 |
| Respiratory quotient | 0.787 | 0.778 | 0.007 | 0.55 |

^1^: Dry matter intake of two days during the methane measuring period.

^2^: Fat-corrected milk in a week before the methane measuring period.

| **Table S5.** Comparison of rumen fermentation parameters between Jersey and Holstein dairy cows | | | | | | | | |
| --- | --- | --- | --- | --- | --- | --- | --- | --- |
| Item | 0 h | | 2.5 h | | SEM | *P*-value | | |
|  | Jersey | Holstein | Jersey | Holstein |  | Breed | Time | Breed × Time |
| pH | 6.78 | 6.72 | 7.00 | 6.64 | 0.046 | 0.012 | 0.75 | 0.11 |
| Dissolved gases | | | | | | | | |
| Methane, m*M* | 0.86 | 0.40 | 1.27 | 1.34 | 0.055 | 0.018 | 0.62 | 0.002 |
| Hydrogen, μ*M* | 13.7 | 1.67 | 72.0 | 9.32 | 5.66 | 0.001 | 0.38 | 0.02 |
| Ammonia-N, m*M* | 7.76 | 11.8 | 11.2 | 13.5 | 0.372 | <0.001 | <0.001 | 0.17 |
| Microbial protein, mg/mL | 30.1 | 53.2 | 38.1 | 50.3 | 3.30 | <0.001 | 0.16 | 0.31 |
| Microbial protein to ammonia-N ratio, g/mmol | 3.92 | 5.29 | 2.92 | 4.21 | 0.316 | 0.032 | 0.090 | 0.95 |
| Total volatile fatty acid, m*M* | 108 | 96.7 | 81.0 | 77.0 | 4.34 | 0.338 | 0.006 | 0.63 |
| Acetate to propionate ratio | 3.93 | 2.76 | 3.14 | 4.06 | 0.097 | <0.001 | 0.036 | 0.31 |
| The molar proportion of individual volatile fatty acids, mol/100 mol | | | | | | | | |
| Acetate | 67.8 | 63.4 | 67.7 | 65.0 | 0.412 | <0.001 | 0.25 | 0.22 |
| Propionate | 17.3 | 23.5 | 16.7 | 21.0 | 0.493 | <0.001 | 0.014 | 0.13 |
| Butyrate | 11.4 | 9.44 | 11.7 | 10.4 | 0.209 | <0.001 | 0.073 | 0.33 |
| Others | 3.52 | 3.66 | 3.88 | 3.55 | 0.072 | 0.526 | 0.36 | 0.11 |

**Table S6.** 432 MAG**s** mapping rate of 24 metagenomic samples

| Sample | Mapping rate |
| --- | --- |
| 117 | 53.26% |
| 118 | 28.98% |
| 121 | 27.30% |
| 122 | 43.09% |
| 125 | 46.87% |
| 127 | 55.04% |
| 130 | 45.50% |
| 132 | 51.82% |
| 133 | 54.19% |
| 134 | 44.53% |
| 135 | 33.99% |
| 136 | 48.21% |
| 137 | 31.47% |
| 140 | 61.66% |
| 141 | 26.83% |
| 142 | 39.34% |
| 143 | 48.31% |
| 145 | 37.20% |
| 146 | 34.54% |
| 147 | 31.11% |
| 149 | 41.39% |
| 150 | 37.81% |
| 151 | 34.87% |
| 152 | 24.67% |
| Average | 40.91% |

**Table S7**. Primer sequences for q-PCR of target species.

| Microbial group | Primer sequence (5′→3′) | Reference |
| --- | --- | --- |
| Total Bacteria | Forward: CGGCAACGAGCGCAACCC  Reverse: CCATTGTAGCACGTGTGTAGCC | Denman and McSweeney (2006) |
| Total Methanogens | Forward: GGATTAGATACCCSGGTAGT  Reverse: GTTGARTCCAATTAAACCGCA | Hook et al. (2009) |
| *Methanobrevibacter* | Forward: CCTCCGCAATGTGAGAAATCGC  Reverse: TCWCCAGCAATTCCCACAGTT | Huang et al. (2016) |

Denman SE, McSweeney CS. Development of a real-time PCR assay for monitoring anaerobic fungal and cellulolytic bacterial populations within the rumen. *FEMS Microbiol Ecol*. 2006;58:572-582.

Hook SE, Northwood KS, Wright AD, McBride BW. Long-term monensin supplementation does not significantly affect the quantity or diversity of methanogens in the rumen of the lactating dairy cow. *Appl Environ Microbiol*. 2009;75:374-380.

Huang XD, Martinez-Fernandez G, Padmanabha J, Long R, Denman SE, McSweeney CS. Methanogen Diversity in Indigenous and Introduced Ruminant Species on the Tibetan Plateau. *Archaea*. 2016;2016:5916067.

**Additional Figures**

**
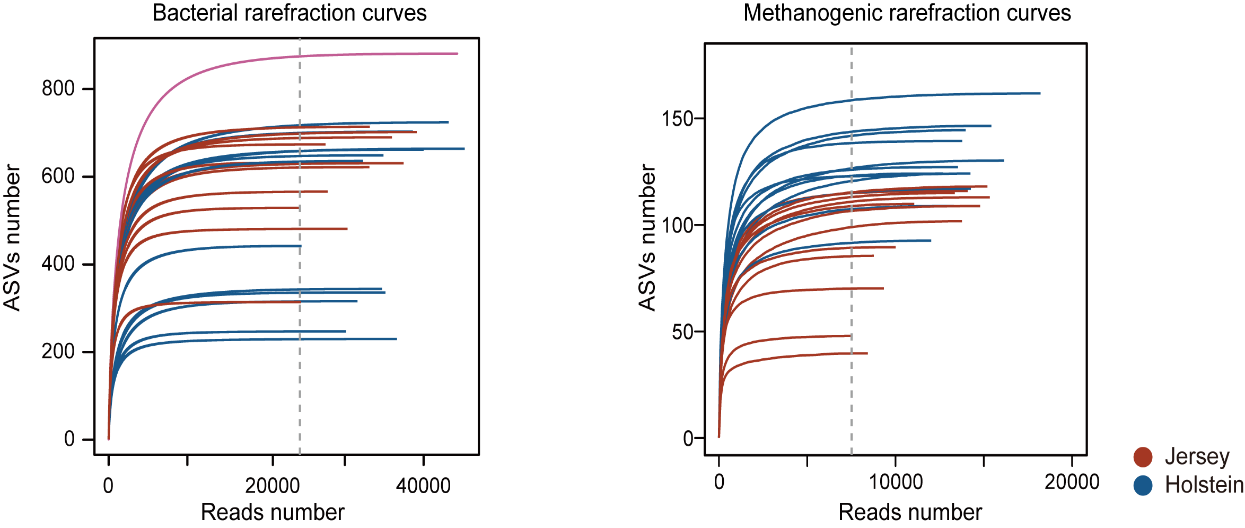
**

**Figure S1. Bacterial and archaeal rarefaction curves based on 16S rRNA gene sequence variants (ASVs).** In all cases, the curves approach asymptotes after bacterial rarefaction curves rarefying to 21,717 and archaeal rarefaction curves rarefying to 8,623, indicating that sequencing depth was adequate to capture the microbial diversity within each sample, *n* = 12/group.

**
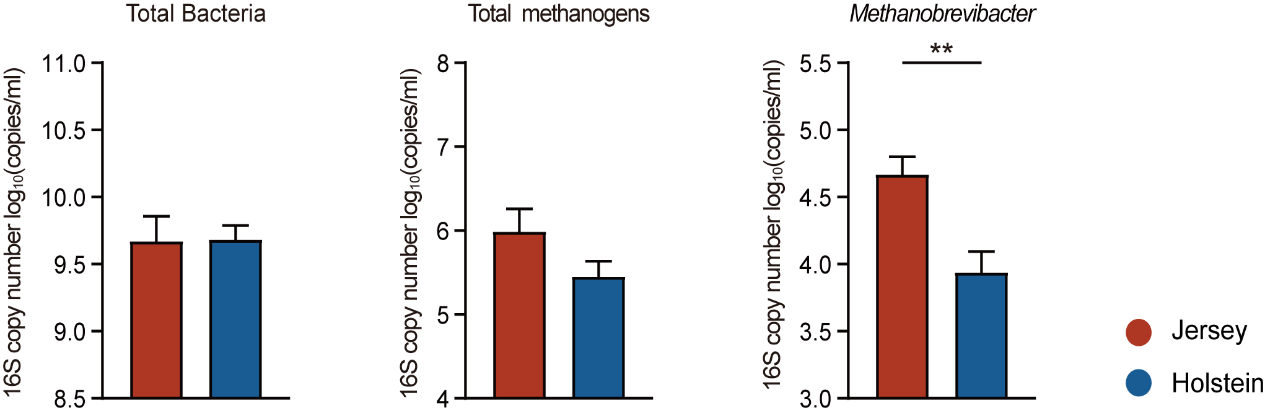
**

**Figure S2. Comparison of 16S rRNA copy numbers of bacteria, methanogens, and genus *Methanobrevibacter* between Jersey and Holstein rumens.** Data with error bars are expressed as mean ± standard error. ***p* < 0.01, n = 12/group.

**
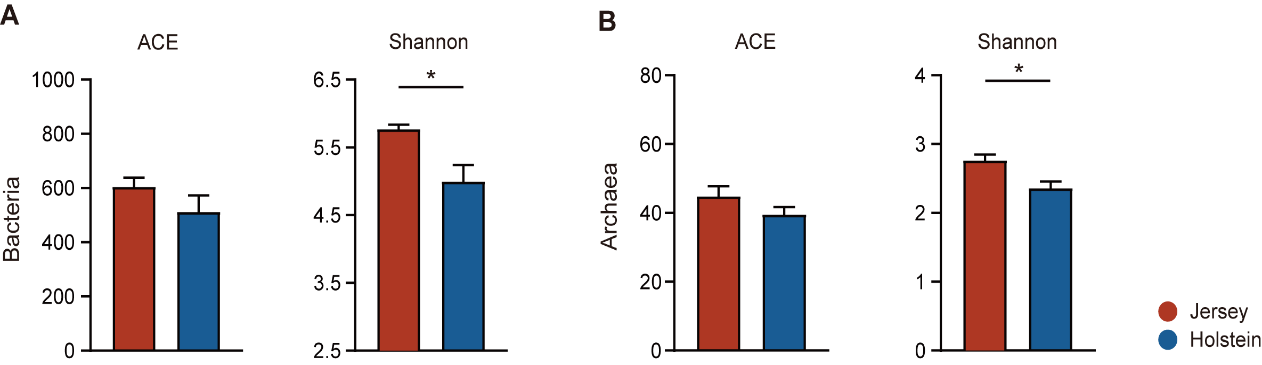
Figure S3. Comparison of alpha (ACE and Shannon index) of bacterial (A) or archaeal community (B) between Jersey and Holstein rumen microbiomes based on 16S rRNA gene amplicon sequence variants (ASV).** Data with error bars are expressed as mean ± standard error. **p* < 0.05, ***p* < 0.01, ****p* < 0.001.


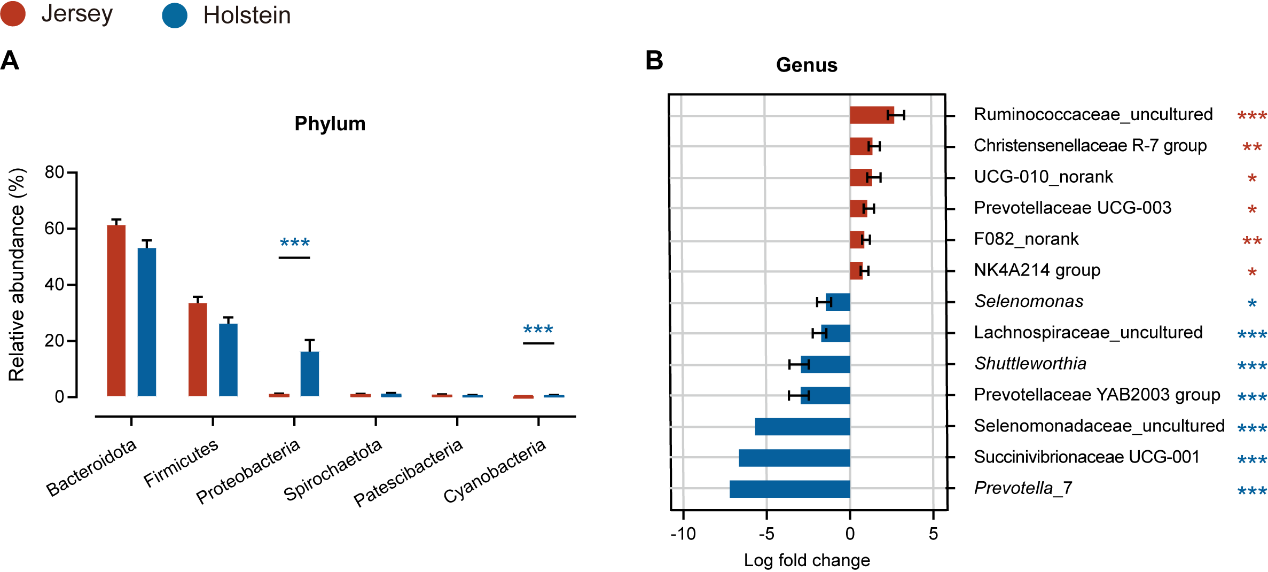


**Figure S4. Comparison of relative abundance of bacterial phyla and genera between Jersey and Holstein rumen microbiomes based on 16S rRNA gene amplicon sequence variants (ASV) using ANCOM-BC analysis.** **A,** Phylum level analyses, only phyla with a relative abundance greater than 0.5% in at least one group were shown; **B,** Genus level analyses, only genera with a relative abundance greater than 0.5% in at least one group and differentiated significantly between the groups were displayed with effect size (log fold change) and 95% confidence error bars. Norank means there is no specific taxonomic information at the genus level. Data with error bars are expressed as mean ± standard error. **p* < 0.05, ***p* < 0.01, ****p* < 0.001, *n* = 12/group.

**
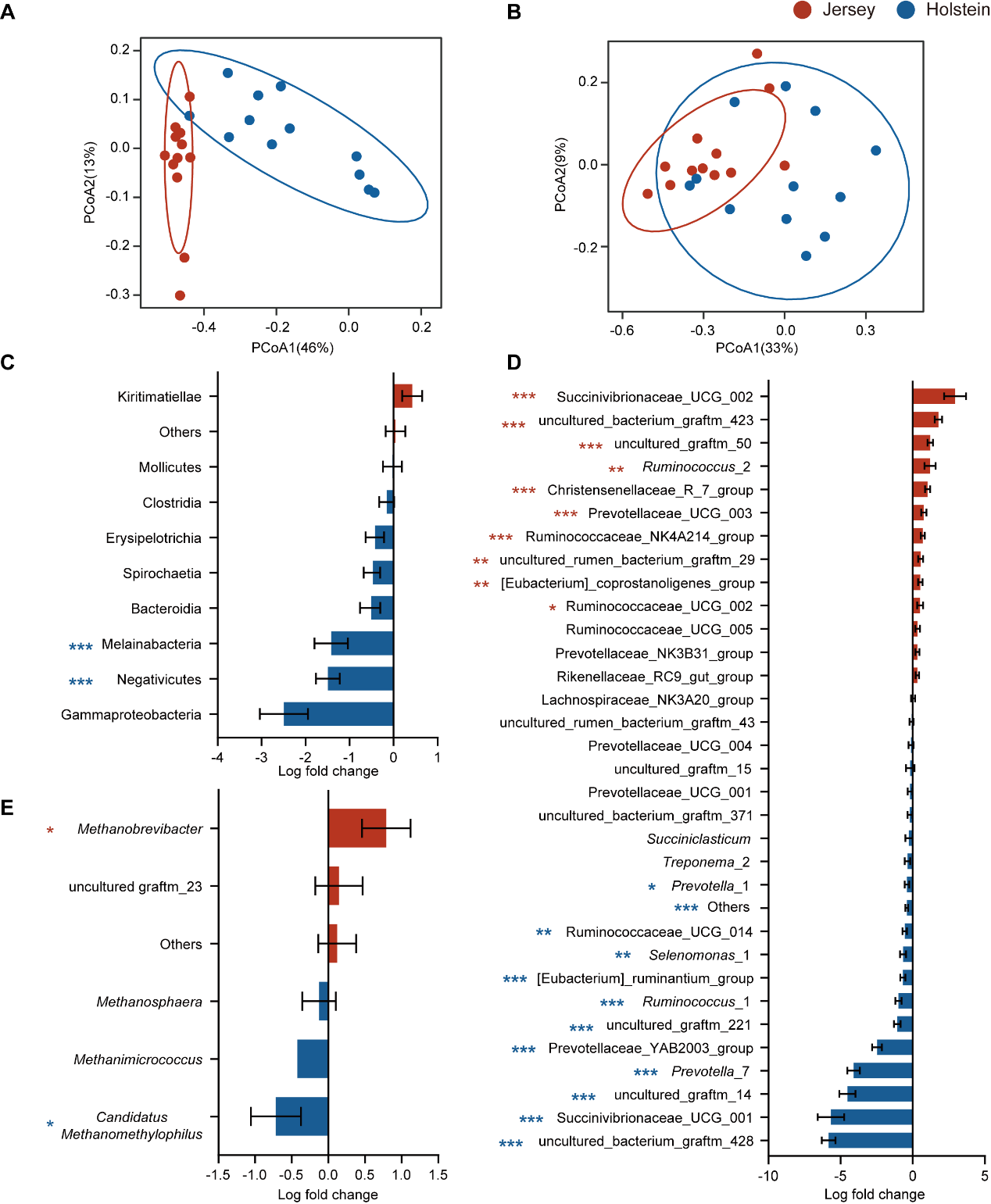
Figure S5. The distinct bacterial and archaeal community between Jersey and Holstein rumen based on metagenome.** Putative 16S sequence fragments were retrieved using GraftM and then aligned to 16S secondary structure in mothur to verify the accuracy of predictions. Sequences successfully aligned were then classified using the naive Bayesian method against the SILVA SSU v132 database. **A,** Principal coordinate analysis profile of bacterial community based on the Bray-Curtis dissimilarity matrix at the operational taxonomic unit level. Testing for significant differences in community structure between breeds was performed using a one-way PERMANOVA with 999 permutations, *p* = 0.001, *R*^2^ = 0.40; **B,** Principal coordinate analysis profile of archaeal community based on Bray-Curtis dissimilarity matrix at the operational taxonomic unit level. Testing for significant differences in community structure between breeds was performed using a one-way PERMANOVA with 999 permutations, *p* = 0.005, *R*^2^ = 0.15; **C,** Bacteria class level analyses; **D,** Bacteria genus level analyses; **E,** Archaeal genus level analyses. Different analyses were based on the ANCOM-BC algorithm. The taxa with average relative abundances less than 0.5% in both groups are not shown and categorized as “others”. **p* < 0.05, ***p* < 0.01, ****p* < 0.001, *n* = 12/group.


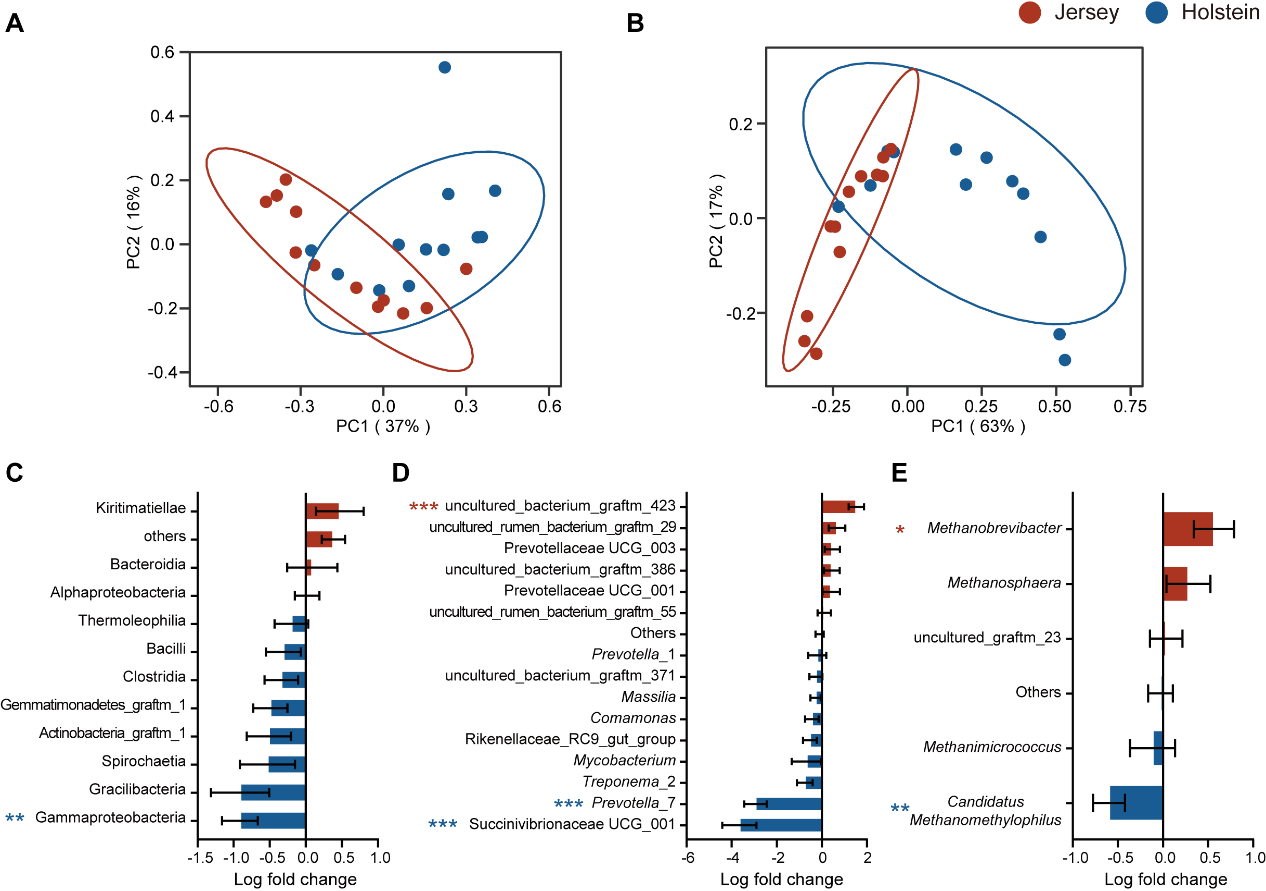


**Figure S6. The distinct bacterial and archaeal community between Jersey and Holstein rumen based on metatranscriptome.** Putative 16S sequence fragments were retrieved using GraftM and then aligned to 16S secondary structure in mothur to verify the accuracy of predictions. Sequences successfully aligned were then classified using the naive Bayesian method against the SILVA SSU v132 database. **A,** Principal coordinate analysis profile of bacterial community based on the Bray-Curtis dissimilarity matrix at the operational taxonomic unit level. Testing for significant differences in community structure between breeds was performed using a one-way PERMANOVA with 999 permutations, *p* = 0.002, *R*^2^ = 0.16; **B,** Principal coordinate analysis profile of archaeal community based on Bray-Curtis dissimilarity matrix at the operational taxonomic unit level. Testing for significant differences in community structure between breeds was performed using a one-way PERMANOVA with 999 permutations, *p* = 0.001, *R*^2^ = 0.34; **C,** Bacteria class level analyses; **D,** Bacteria genus level analyses; **E,** Archaeal genus level analyses. Different analyses were based on the ANCOM-BC algorithm. The taxa with average relative abundances less than 0.5% in both groups are not shown and categorized as “others”. **p* < 0.05, ***p* < 0.01, ****p* < 0.001, *n* = 12/group.


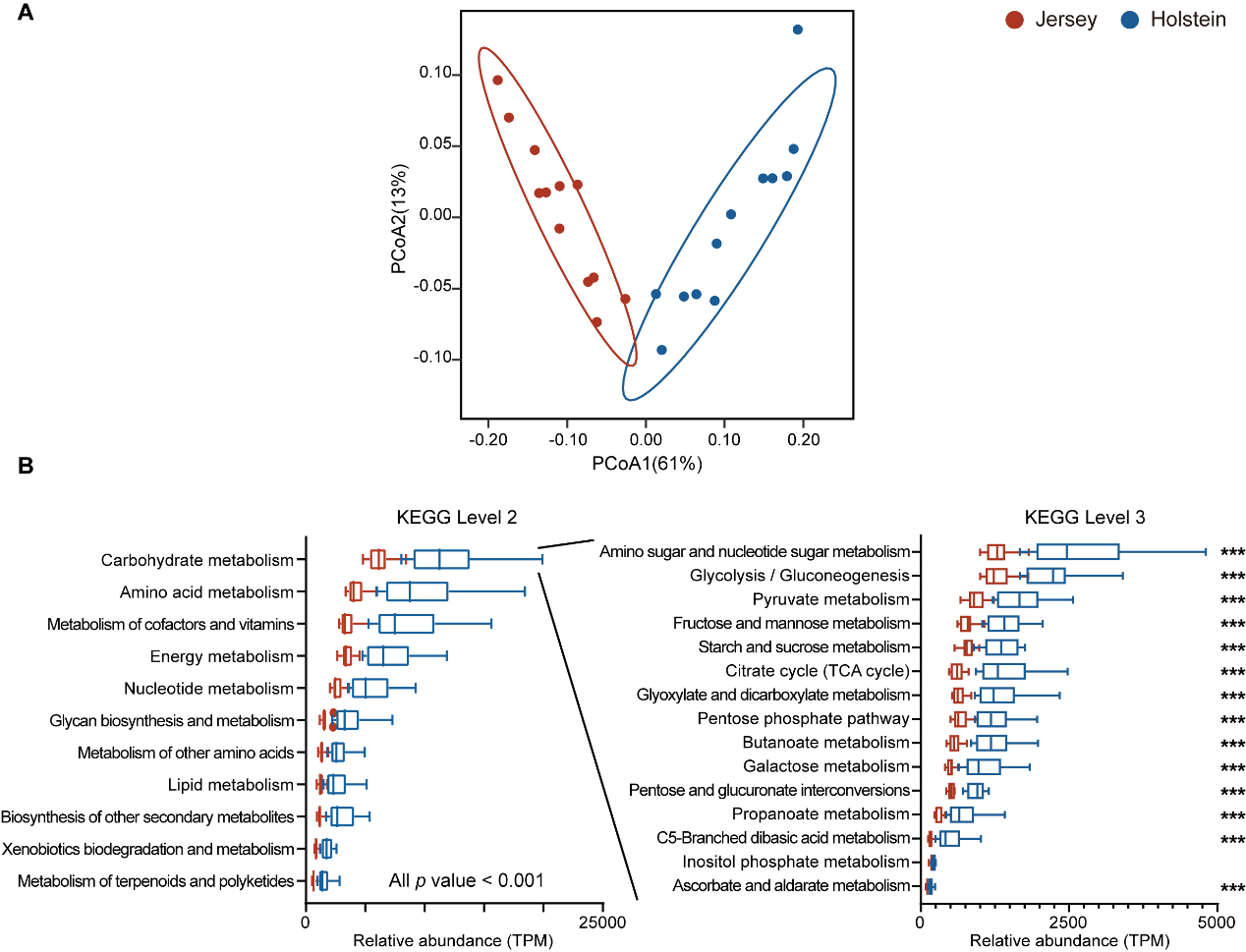


**Figure S7. Comparison of Kyoto Encyclopedia of Genes and Genomes (KEGG) profile between Jersey and Holstein rumen metagenome. A,** Principal coordinate analysis (PCoA) profile of all KO genes. Abundance-based distance matrix Bray-Curtis diversity was employed, and testing for significant differences in community structure between breeds was performed using a one-way PERMANOVA with 999 permutations, *p* = 0.001, *R*^2^ = 0.31; **B,** The relative abundance of genes at KEGG level-2 and level-3 pathways. Whiskers represent the lowest and highest values. ****p* < 0.001 *n* = 12/group.


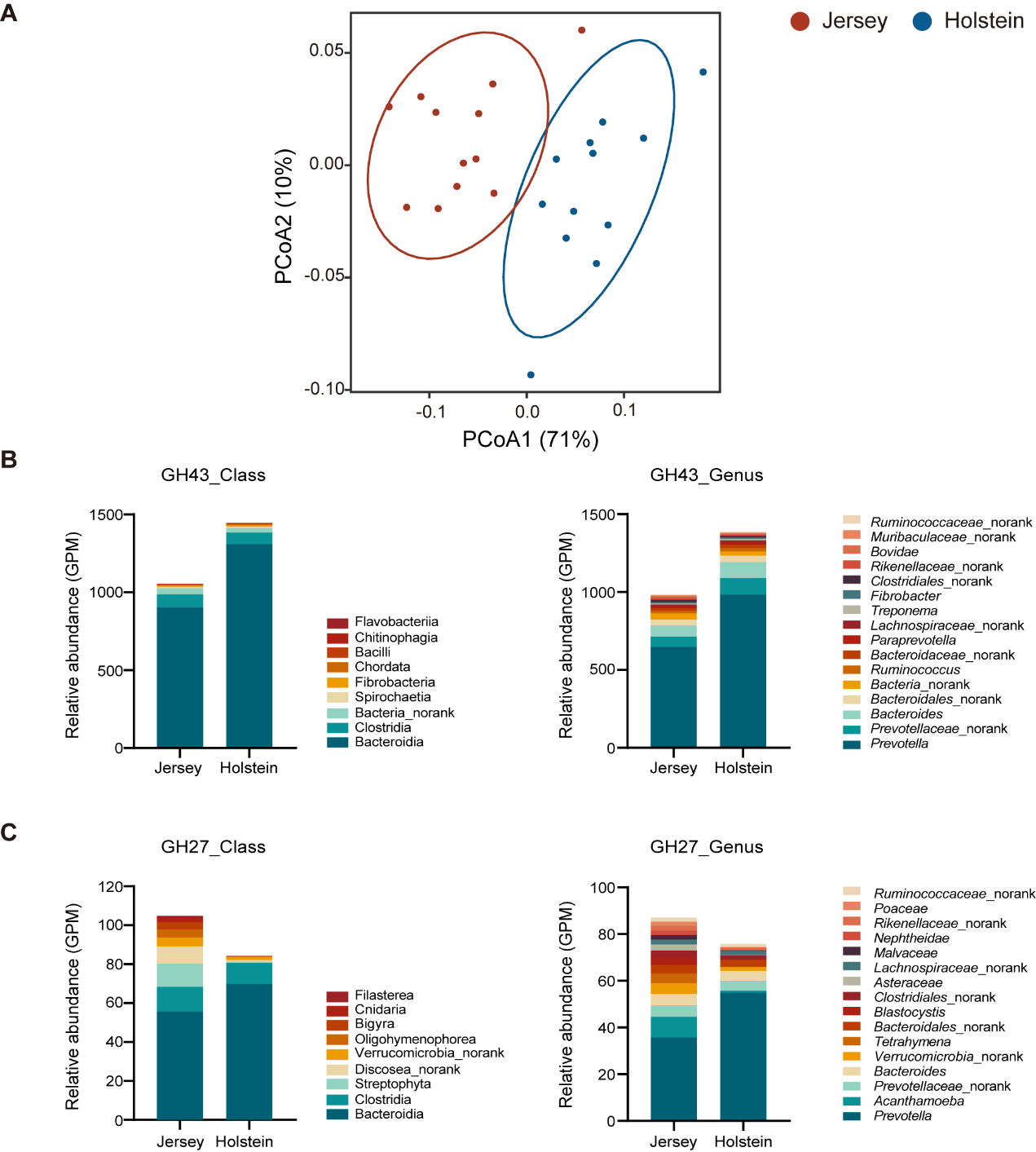


**Figure S8. Comparison of CAZymes profile between Jersey and Holstein-rumen metagenome. A,** PCoA profile of all CAZy families. Abundance-based distance matrix Bray-Curtis diversity was employed, and testing for significant differences in community structure between breeds was performed using a one-way PERMANOVA with 999 permutations, *p* = 0.001, *R*^2^ = 0.49; B, Phylogenetic distribution of GH43 assigned to the identified class and genus in Holstein and Jersey rumen microbiome; C, Phylogenetic distribution of GH27 assigned to the identified class and genus in Holstein and Jersey rumen microbiome. n = 12/group.


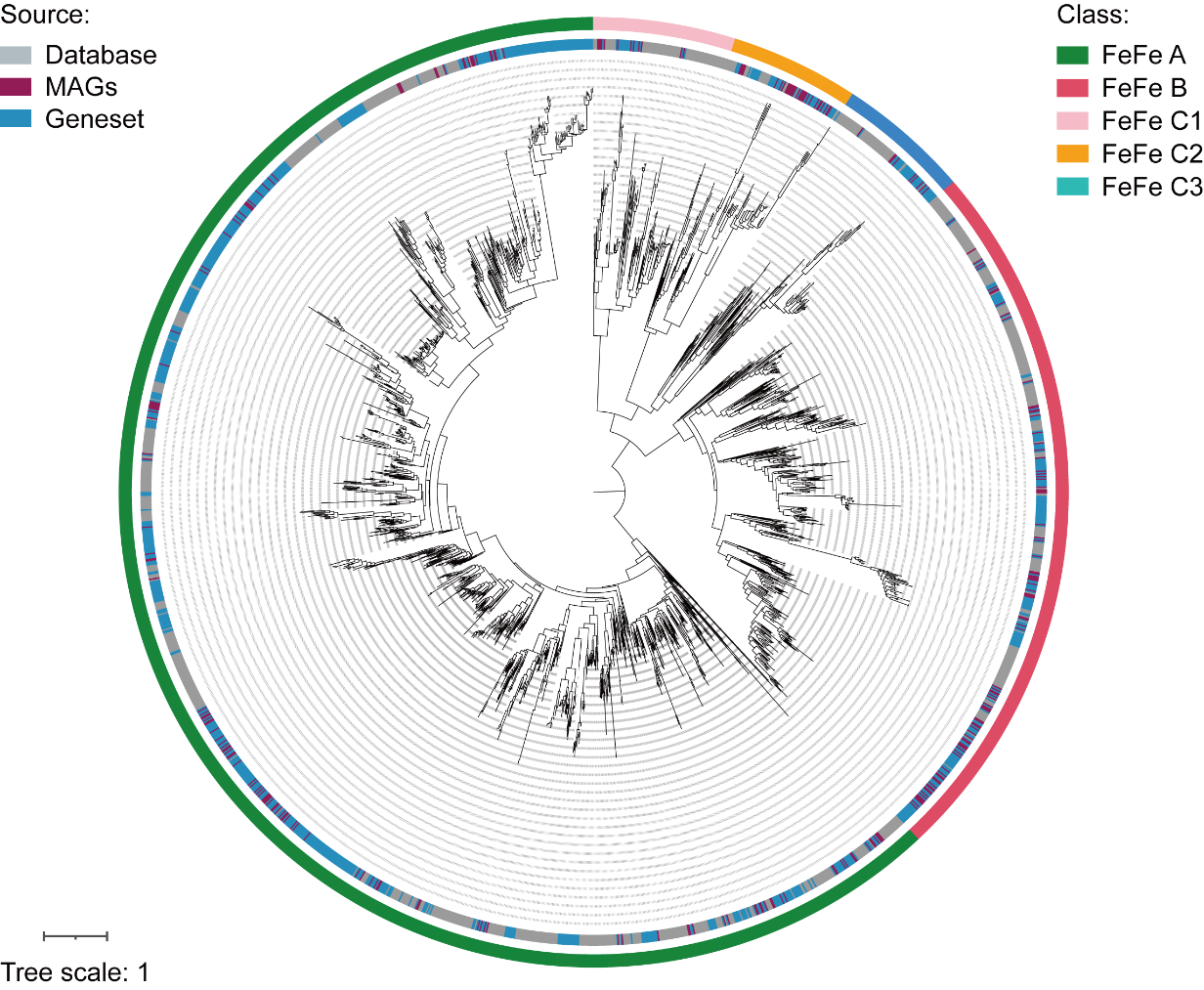


**Figure S9. Maximum-likelihood phylogenetic tree of amino acid sequences of FeFe hydrogenases.** The tree shows sequences from metagenome-assembled genomes (purple) and unbinned contigs (blue) alongside representative reference sequences (grey). The different color in the outer layer shows the corresponding sequence's hydrogenase class. The tree was constructed using the JTT matrix-based model and was bootstrapped with 1,000 replicates and midpoint-rooted.


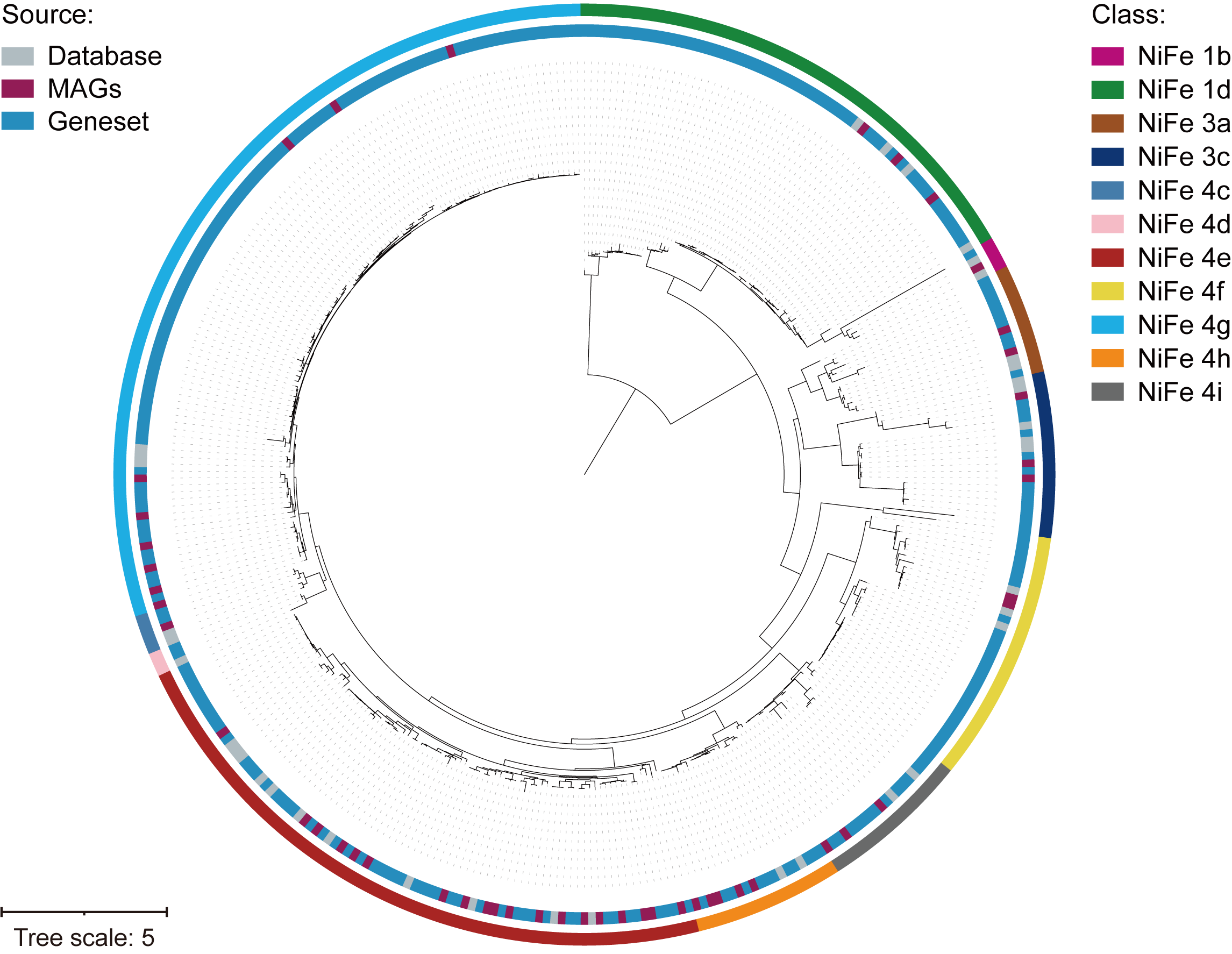


**Figure S10. Maximum-likelihood phylogenetic tree of amino acid sequences of NiFe hydrogenases.** The tree shows sequences from metagenome-assembled genomes (purple) and unbinned contigs (blue) alongside representative reference sequences (grey). The different color in the outer layer shows the corresponding sequence's hydrogenase class. The tree was constructed using the JTT matrix-based model and was bootstrapped with 1,000 replicates and midpoint-rooted.


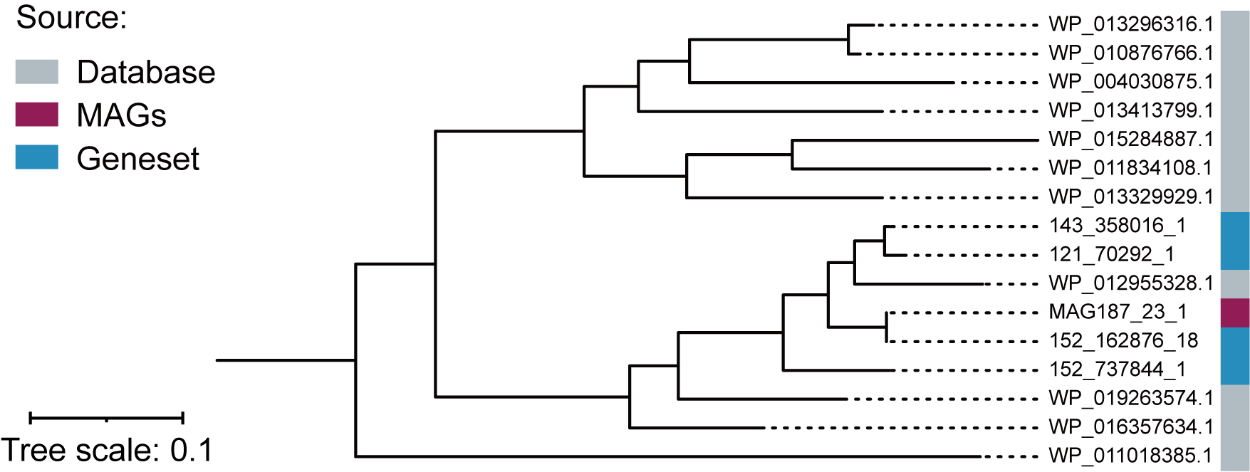


**Figure S11. Maximum-likelihood phylogenetic tree of amino acid sequences of Fe hydrogenases.** The tree shows sequences from metagenome-assembled genomes (purple) and unbinned contigs (blue) alongside representative reference sequences (grey). The tree was constructed using the JTT matrix-based model and was bootstrapped with 1,000 replicates and midpoint-rooted.


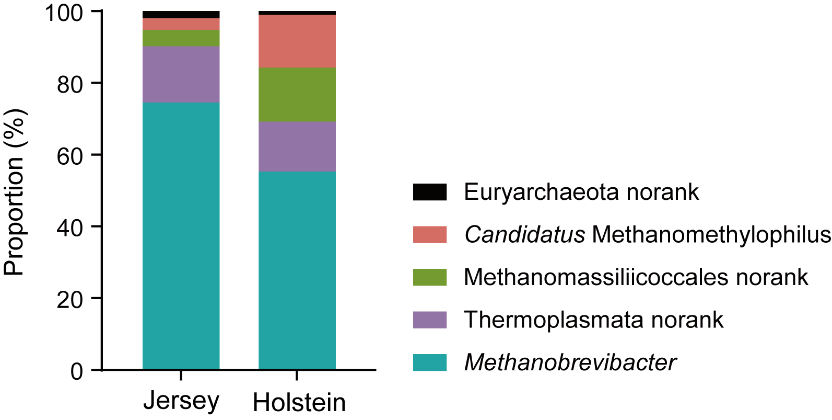


**Figure S12. Phylogenetic distribution of *mcrA* genes in Jersey and Holstein cows at genus level based on metagenomes.**


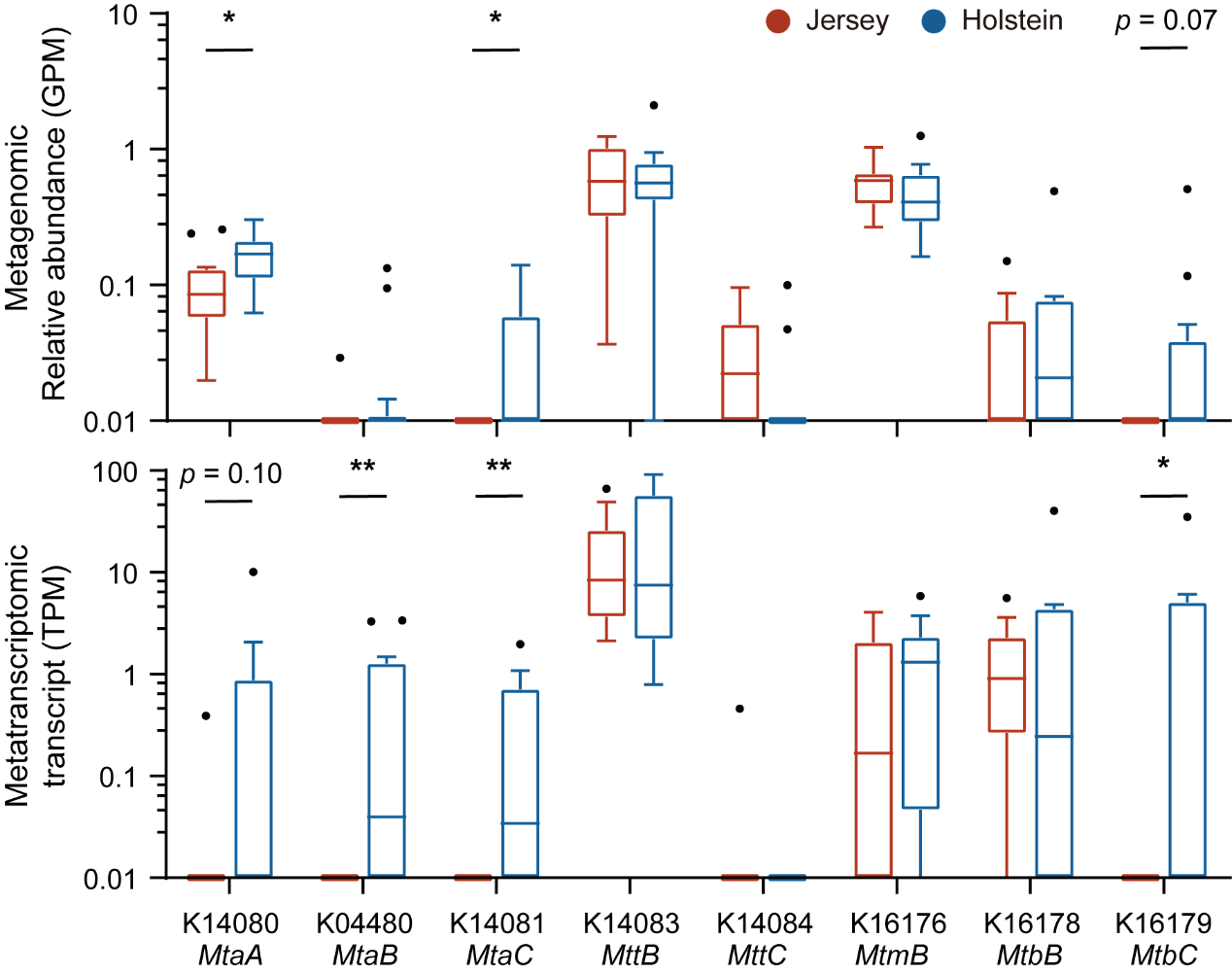


**Figure S13. Comparison of metagenomic relative abundance and metatranscriptomic transcript of methyl-transferase key enzymes.** *P* values between 0.05 and 0.1 were considered to have a trend towards significance and were labeled. **p* < 0.05, ***p* < 0.01, ****p* < 0.001, *n* = 12/group.


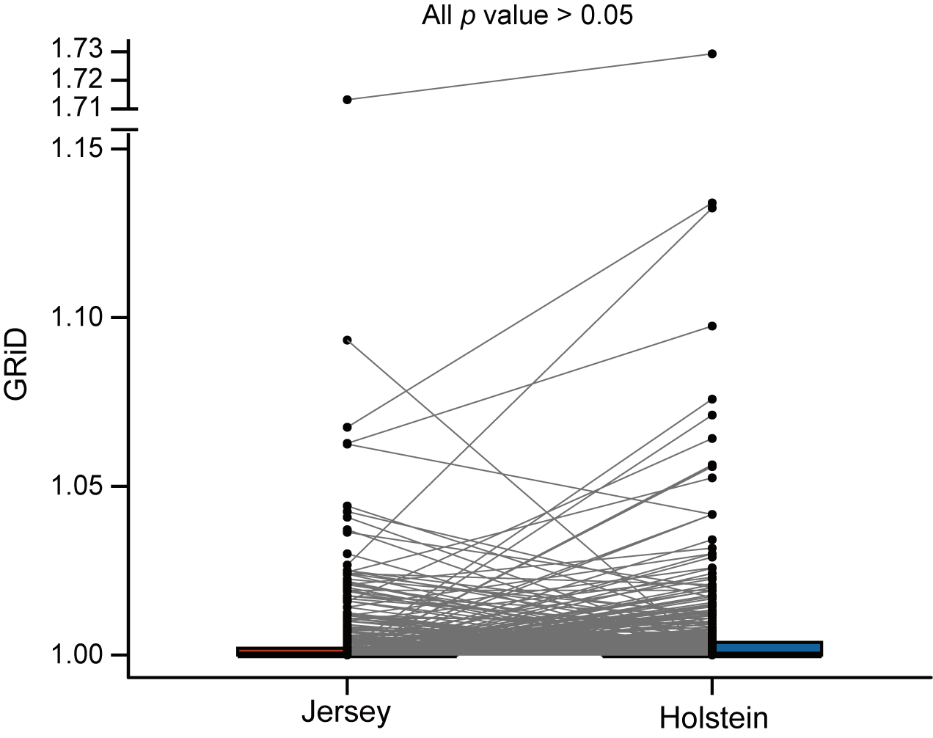


**Figure S14. Comparison of Growth Rate InDex (GRiD) of MAGs between Jersey and Holstein-rumen metagenome.** Each point represents the average GRiD value of each MAG in the corresponding group, and solid lines connect the same MAGs. Only MAGs that coverage exceed the threshold are displayed.


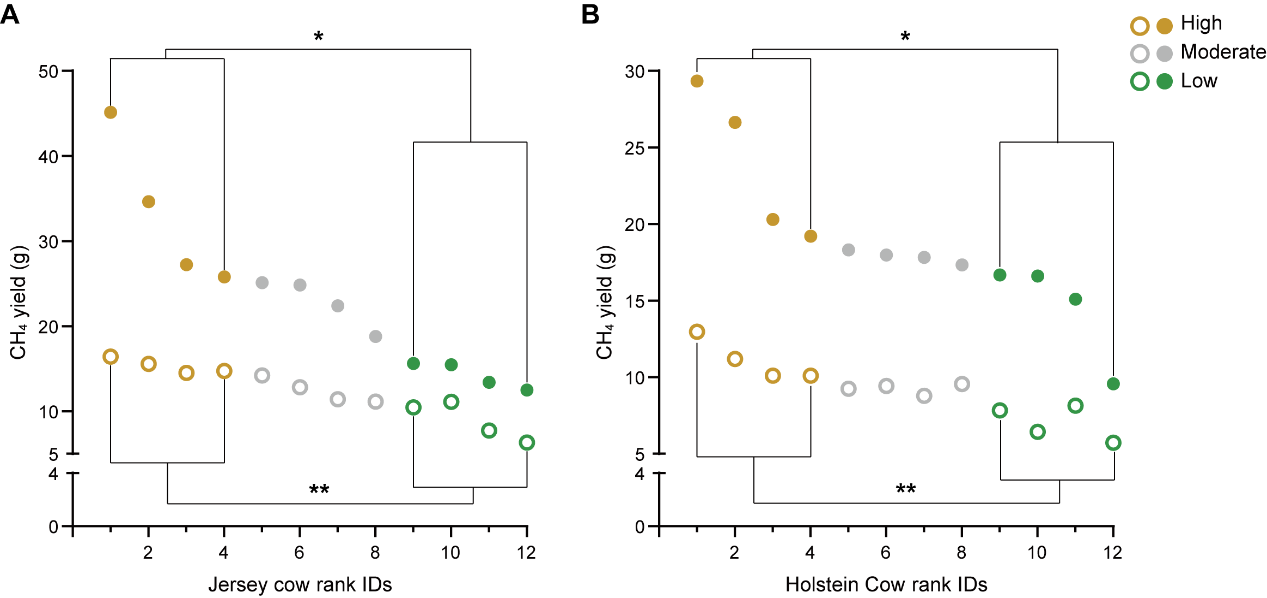


**Figure S15. The selection of four high- and four low-methane-emitters from total 12 Jersey (A) and Holstein dairy cows (B) respectively.** The cow ranks according to the descending of methane (CH_4_) yield. Solid dots, CH_4_ yield corrected for dry matter intake; hollow dots, CH_4_ yield corrected for fat-corrected milk production; **p* < 0.05, ***p* < 0.01.

**Supplementary Materials and Methods**

**Rumen sample collection and analysis**

Feed intake was recorded daily for each cow. Fecal samples were collected from the rectum for proximate composition analysis [1] and used to calculate apparent nutrient digestibility using acid-insoluble ash as an endogenous marker [2]. Rumen samples were collected at 0 and +2.5 h relative to the commencement of the morning feeding using a stomach tube, with the first 150 mL of rumen contents discarded to avoid saliva contamination [3]. About 20 mL of sampled rumen content was immediately used to measure pH using a portable pH meter (Starter 300; Ohaus Instruments Co. Ltd., Shanghai, China). Three 50-mL subsamples were snap-frozen in liquid N_2_ and stored at -80 °C for DNA and RNA extraction. Two 35-mL subsamples were immediately transferred to 50-mL plastic syringes for measuring dissolved gas concentrations [4]. Three 5-mL subsamples of rumen contents were collected and centrifuged at 12,000 × *g* for 10 min at 4 °C. A 1.5 mL aliquot of the supernatant was acidified with 0.15 mL of meta-phosphoric acid (25%, w/v) and stored at -20 °C for subsequent measurement of fermentation products.

Ruminal dissolved hydrogen (dH_2_) and methane (dCH_4_) were firstly extracted from the liquid phase of rumen contents into gaseous nitrogen of syringes using the procedure by Wang et al. (2014) [4]. Gases composition was measured using a gas chromatograph (Agilent 7890A, Agilent Inc., Palo Alto, CA) [3] and used to calculate ruminal dH_2_ and dCH_4_ concentrations using the equations by Wang et al. (2016) [3]. Ammonia-N concentration was determined by Weatherburn et al. (1967) [5]. Microbial cells in rumen fluid were separated by differential centrifugation according to Makkar et al. [6], and the microbial protein concentration in rumen fluid was measured colorimetrically according to Bradford protein assay [7] using a Coomassie brilliant blue kit (Build a biopharmaceutical research institute, Nanjing, China). Individual volatile fatty acids (VFA) concentrations were analyzed by gas chromatography described by Wang et al. (2014) [4].

Targeted metabolomics of central carbon metabolism in rumen liquid samples was conducted by positive/negative Multiple Reaction Monitoring (MRM) in Novogene Co., Ltd. (Beijing, China). The samples (100 μL) were homogenized with 500 μL of methanol (80%) and centrifuged to remove the protein. The supernatant was added to the derivatization reagent (50 μL) and derivatized at 40℃ for 40 min. Then, the supernatant (100 μL) was homogenized with 1 μL mixed internal standard solution. They were finally injected into the LC-MS/MS system for analysis. An ultra-high performance liquid chromatography coupled to tandem mass spectrometry (UHPLC-MS/MS) system (Vanquish™ Flex UHPLC-TSQ Altis™, Thermo Scientific Corp., Germany) was employed. Separation was performed on a Waters HSS T3 column (2.1×150 mm) maintained at 40 °C. The mobile phase, consisting of 0.1% formic acid in water (solvent A) and acetonitrile (solvent B), was delivered at a flow rate of 0.40 mL/min. The solvent gradient was set as follows: initial 40% B; 1 min, 40% B; 3.5 min, 40-60% B; 8.5 min, 60-75% B; 11.5 min, 75-100% B; 16 min; 100-60% B. The mass spectrometer was operated in positive/negative multiple reaction (MRM) mode. Parameters were as follows: IonSpray Voltage (3500V/-2500 V), Sheath Gas (35 psi), Ion Source Temp (350 °C), Auxiliary Gas (60 psi), Collision Gas (1.5 psi). LC-MS was used to detect the concentration series of standard solution. The ratio of the concentration of standard to internal standard was as abscissa, and the ratio of peak area of standard to internal standard was as ordinate to investigate the linearity of standard solution. The necessary condition was the correlation coefficient (r) > 0.99 of each metabolite. The limit of quantification (LOQ) was determined by the signal-to-noise ratio (S/N) method, which compares the signal measured by the standard solution concentration with the blank matrix.

**Microbial DNA and RNA extraction**

Microbial DNA was extracted following the protocol by Ma et al. [8] based on repeated sand beating plus column methodology [9] and purified by phenol/chloroform/isopentyl alcohol (25:24:1 vol/vol/vol, Solarbio Co., Shanghai, China). The integrity of the extracted DNA was assessed by electrophoresis on 0.8% agarose gels, and DNA concentration and quality were determined using an ND-2000 spectrophotometer (NanoDrop Technologies, Wilmington, DE). All DNA samples were stored at −80 °C until subsequent analyses.

Microbial RNA was extracted from the same batch of rumen contents and was isolated using the TRIzol^®^ Reagent (Invitrogen) according to the manufacturer’s instructions. Genomic DNA was removed using DNase I (TaKara). RNA quality was determined using 2100 Bioanalyser (Agilent), and RNA was quantified using an ND-2000 spectrophotometer (NanoDrop Technologies).

**Quantitative RT-PCR Analyses**

Quantifying bacteria, methanogenic archaea, and the genus *Methanobrevibacter* was conducted by quantitative real-time PCR (qPCR) with primers validated in our laboratory (additional Table S5). The qPCR assays were performed according to the procedures described by Ma et al. [10] using a LightCycler 480 (Roche Molecular Systems Inc., Pleasanton, CA) and qPCR SuperMix (Transgen, Beijing, China). Final absolute amounts of target groups were estimated by relating the cycle threshold value to standard curves and expressed as copies per milliliter of rumen fluid (log_10_ transformed).

**Metagenome sequencing, assembly, and binning**

All samples were sequenced on the HiSeq X platform (Illumina, San Diego, CA, USA) with pair-end 150 bp (PE150) mode at Shanghai Biozeron Biological Technology Co. Ltd. Subsequently, low-quality reads, contaminated adaptors and host reads (Bos_taurus. UMD3.1 [11] and hg19 [12]) were discarded using the Trimmomatic (http://www.usadellab.org/cms/?page=trimmomatic) and BWA packages (v.0.7.12) [13]. An average of 13.5 gigabases of paired-end reads per sample was obtained for further analysis. MEGAHIT (v.1.1.1) was used to assemble the clean reads with “-min-contig-len 500” parameters [14]. Prodigal (version 2.6.3) [15] was used to predict the contigs from each sample with ‘-meta’ parameters, and the ORFs derived from assembled contigs were maintained and clustered into a nonredundant data set by CD-HIT (v.4.8.1, parameter: -n 9 -g 1 -c 0.95 -G 0 -M 0 -d 0 -aS 0.9) [16]. A pan-metagenome was constructed and used to analyze the subsequent predicted metagenomic function of the rumen microbiome. Original sequences were mapped to predicted genes to estimate the abundances using Salmon software with default parameters [17]. The abundance profile of genes (alignment length ≥ 50 bp and sequence identity > 95%) was calculated and transformed to gene per million (GPM) [18] and to transcripts per million (TPM) for RNAseq analysis [19], with corrections for variations in gene length and mapped reads per sample.

Metagenomic binning was performed in each sample contig to obtain metagenome-assembled genomes (MAG). We used metaBAT2 [20] (v.2.12.1) to perform separate binning. The completeness and contamination of all bins were verified with CheckM [21] (v.1.1.2). All bins with completeness >50% and contamination <10% were considered as “filtered bins”. All MAGs were dereplicated with a 99% ANI cutoff using dRep [22] (v.2.5.4; parameter: -p 72 --ignoreGenomeQuality -pa 0.95 -sa 0.99) to obtain 432 nonredundant MAGs. All genomes were taxonomically annotated by GTDB-Tk (v.1.3.0) [23] based on the Genome Taxonomy Database (http://gtdb.ecogenomic.org/). High-quality reads in each sample were aligned against each assembled genome using BWA-MEM [23] (v.0.7.17), and the relative abundance of each MAG in each sample was calculated according to the GPM calculation process. The GRiD algorithm (v.1.3) was used to estimate the growth rates of each MAG by calculating the coverage in replication origin and terminal origin[24]. The correlation network was constructed using Gephi [25] (v.0.9.2) based on the key genes involved in pathways of hydrogen metabolism, under the assumption of a connecting line between MAG and metabolism, with MAG encoding the corresponding metabolic genes. Integrated public MAGs were created using the superset database built by Stewart et al. [26] and the rumen MAGs published by Xie et al. [27]. Under de-replication with a 99% ANI cutoff using dRep, 7,651 public rumen MAGs were obtained for further analysis.

The taxonomy of metagenomic clean reads for each sample was generated with GraftM software [28] (v.0.14.0) using the 7.71.silva_v132_alpha1.gpkg reference package. To estimate the gene abundance in the microbial community, high-quality unassembled reads were also screened for the 14 universal single-copy ribosomal marker genes used in SingleM (v.0.13.2) (https://github.com/wwood/singlem). The proportion of community members that encode each gene was estimated by dividing the read counts for the gene (in reads per kilobase million [RPKM]) by the mean of the read counts of 14 universal single-copy ribosomal marker genes (in RPKM).

**Biodiversity analysis**

Alpha diversity of samples, including ACE and Shannon indexes, was evaluated using MOTHUR v.1.39 [29]. Principal coordinate analysis (PCoA) was performed to reveal the differences between samples using the *vegan* package [30] R based on the Bray-Curtis dissimilarity matrix. Analysis of similarities (ADONIS) was performed to indicate group similarity, and the *p* values were determined based on 999 permutations.

**Functional annotation**

To compare the metabolic capability of rumen microbial communities, metagenomes, metatranscriptomes, and MAGs were searched against a local protein database of representative metabolic genes related to reductant disposal reactions involved in carbohydrate degradation and amino acids synthesis, molecular hydrogen (H_2_) production and incorporation, and marker genes for key metabolic processes. Searches were carried out using all quality-filtered genes through a combination of hidden Markov models (HMMs) and homology-based searches. Genes were also annotated against the KEGG database for analyzing carbohydrate metabolism by HMM searches with default parameters and then summarized through the abundance of level 3 and level 2 pathways, along with KOs involved in VFA production [31].

Entries in all the gene sets were subjected to the taxonomic and functional assignment using DIAMOND based on BLASTP searches against the NCBI-NR (October 2018; approximately 550 M sequences). The gene set was also aligned with the CAZy database (<http://www.cazy.org/>) [32] using HMMER [33](v.3.3.1) to harvest corresponding annotations for CAZyme.

**Phylogenetic analysis**

Functional protein sequences retrieved from MAGs and assembled unbinned reads by homology-based searches were aligned against a subset of reference sequences from the custom protein database using MUSCLE [34] (v.3.8.1551). Trees were constructed using FastTree [35] (v.2.1.10) through the maximum-likelihood method and visualized by iTOL [36] (v.6.6).

To determine the phylogenic affiliation and diversity of the MAGs, PhyloPhlAn (v.3.0.58) was applied to build a phylogenetic tree by aligning the individual proteins from the protein sets recovered from the input genomes using MUSCLE. The most discriminative positions in each protein alignment were concatenated into one single long sequence, which was used to reconstruct a maximum-likelihood tree through FastTree. Finally, the phylogenetic tree was visualized by iTOL.

**Measurements of microbiome activity through *in vitro* experiments**

The first *in vitro* experiment (“*In vitro* Experiment 1”) was conducted to compare the fermentative activities, including gases and VFA production, of Jersey and Holstein rumen microbiome, by incubating the TMR fed to the animals according to the procedure of Wang et al. (2016) [37]. Jersey and Holstein rumen contents were collected from all the animals in the study before the morning feeding using a stomach tube and taken to the laboratory. All laboratory procedures were performed under a stream of CO_2_. For each bottle, rumen content was separately filtered through four layers of cheesecloth and then mixed with pre-warmed McDougall's buffer (volume ratio of 1 to 4) to prepare the buffered rumen fluid. Buffered rumen fluid (60 mL) was delivered into 150-ml bottles containing 1 g substrate and sealed. *In vitro* batch cultures were incubated at 39.5 ℃ for 48 h. The pressure inside each bottle was measured and recorded every 1 min. When the pressure inside any bottle exceeded 10 kPa, the three-way solenoid valve on that bottle opened to release the excess gas, and CH_4_ and H_2_ concentrations were determined through gas chromatography (Agilent 7890A, Agilent Inc., Palo Alto, CA) [3]. Methane production was then calculated using the equation of Wang et al. (2013). Samples from the liquid phase of the bottle were collected from each bottle after the incubations, snap-frozen in liquid N_2_, and stored at -80 °C for further analysis of fermentation end products.

The second *in vitro* experiment (“*In vitro* Experiment 2”) was conducted to compare the activities of Jersey and Holstein rumen microbiomes to use H_2_ by adding molecular deuterium (^2^H_2_ or D_2_) to the incubation bottle headspace. The blank control (BC) contained McDougall's buffer without inoculum. Jersey and Holstein rumen contents were collected from animals before the morning feeding using a stomach tube and taken to the laboratory. All laboratory procedures were performed under a stream of CO_2_. Rumen contents were filtered through five layers of cheesecloth and then mixed with pre-warmed McDougall's buffer (volume ratio of 1 to 9). Buffered rumen fluid (30 mL) was delivered into 150-mL bottles, while 7 mL 1% ²H_2_ gas was added into the headspace of the bottle to reach a concentration of 580 ppmv, which is within the range of gH_2_ concentration in the rumen headspace [38, 39]. Samples of each bottle headspace (2 mL) were collected at 1, 4, 8, 12, 24, 36, and 48 h of incubation and used to measure headspace H_2_ and CH_4_ concentrations through gas chromatography (Agilent 7890A, Agilent Inc., Palo Alto, CA) [3]. The batch culture was incubated for 48 h at 39.5 ℃. At the end of the incubation, samples from the liquid phase were collected from each bottle to analyze for ²H enrichment in fermentation end products.

**Isotope ratio measurement**

The C and H isotopic composition of individual VFA was measured using GC- IsoLink -MS/IRMS system [40]. The GC-IsoLink-MS/IRMS system consisted of a Trace GC Ultra equipped with a TriPlus autosampler, retrofitted to the interface GC IsoLink II for C/H (Thermo Scientific, Bremen, Germany), hyphenated to a Delta V Advantage isotope ratio mass spectrometer (Thermo Scientific, Bremen, Germany), with a single-quadrupole GC–MS (ISQ Thermo Scientific, Milan, Italy). Individual VFA were firstly separated using a GC- IsoLink -MS module equipped with a DB-FFAP 30 m × 0.25 mm i.d., 0.25 μm film thickness, capillary column (Agilent, Santa Clara, CA, United States), with helium as carrier gas at a flow of 1.2 mL/min. The programmed operating temperature for analysis was as follows: 60 °C held for 2 min and then ramped to 220 °C at 20 °C/min, and held at 220 °C for 0.5 min. The injection was performed at 250 °C in splitless mode (2 min) with an injection volume of 1 μL.

The ion source temperature was 230 °C, and the quadruple was 150 °C. The mass spectrometer was operated at 70 eV, and the scan range was 50-500 m/z. Individual VFA were identified through their retention times as compared against reference standards and compared with the NIST library (NIST Standard Reference Database1 A NIST/EPA/NIH Mass Spectral Library (NIST 17) and NIST Mass Spectral Search Program (Version 2.2f). Data were collected using the Isodat 3.0 software (Thermo Fisher Scientific).

For C isotopic ratio analysis, the separated samples were transported to the interface GC IsoLink II at C module and passed through a combustion reactor at 900 °C to oxidize the VFA C to CO_2_ gas, and the C isotopic composition of the CO_2_ produced was analyzed by IRMS. For H isotopic composition analysis, samples were passed through a pyrolysis reactor at 1400 °C to convert the H in VFA to H_2_ gas. Before measuring the ^2^H/^1^H ratio, the [H_3_]^+^ factor was verified to be lower than 8. It was found to be generally around 5, with a maximum daily variation of 0.05. The H isotopic composition of the H_2_ produced was analyzed by IRMS. The C and H isotopic composition for all standards and samples was normalized according to Vienna Pee Dee Belemnite (VPDB) and Vienna Standard Mean Ocean Water (VSMOW), respectively. Both carbon and hydrogen isotopic composition were reported in the delta notation and calculated as follows:

δ^13^C or δ^2^H = 10^3^(R_sample_/R_standard_ -1)

R= ^13^C/^12^C or ^2^H/^1^H of the sample and the standard, respectively.

**Reference:**

1. AOAC, Horwitz W. Official methods of analysis of AOAC International. 16th ed. Arlington, VA: Association of Official Analytical Chemists; 1995.

2. Van Keulen J, Young BA. Evaluation of acid-insoluble ash as a natural marker in ruminant digestibility studies. *J Anim Sci*. 1977;44:282-287.

3. Wang M, Wang R, Janssen PH, Zhang XM, Sun XZ, Pacheco D, et al. Sampling procedure for the measurement of dissolved hydrogen and volatile fatty acids in the rumen of dairy cows. *J Anim Sci*. 2016;94:1159-1169.

4. Wang M, Sun XZ, Janssen PH, Tang SX, Tan ZL. Responses of methane production and fermentation pathways to the increased dissolved hydrogen concentration generated by eight substrates in *in vitro* ruminal cultures. *Anim Feed Sci Technol*. 2014;194:1-11.

5. Weatherburn MW. Phenol-Hypochlorite reaction for determination of ammonia. *Anal Chem*. 1967;39:971-974.

6. Makkar HPS, Sharma OP, Dawra RK, Negi SS. Simple determination of microbial protein in rumen liquor. *J Dairy Sci*. 1982;65:2170-2173.

7. Bradford MM. A rapid and sensitive method for the quantitation of microgram quantities of protein utilizing the principle of protein-dye binding. *Anal Biochem*. 1976;72:248-254.

8. Ma ZY, Zhang XM, Wang R, Wang M, Liu T, Tan ZL. Effects of chemical and mechanical lysis on microbial DNA yield, integrity, and downstream amplicon sequencing of rumen bacteria and protozoa. *Front Microbiol*. 2020;11:581227.

9. Yu ZT, Morrison M. Improved extraction of PCR-quality community DNA from digesta and fecal samples. *Biotechniques*. 2004;36:808-812.

10. Ma Z, Wang R, Wang M, Zhang X, Mao H, Tan Z. Short communication: Variability in fermentation end-products and methanogen communities in different rumen sites of dairy cows. *J Dairy Sci*. 2018;101:5153-5158.

11. Shamimuzzaman M, Le Tourneau JJ, Unni DR, Diesh CM, Triant DA, Walsh AT, et al. Bovine Genome Database: new annotation tools for a new reference genome. *Nucleic Acids Res*. 2020;48:676-681.

12. International Human Genome Sequencing C. Finishing the euchromatic sequence of the human genome. *Nature*. 2004;431:931-945.

13. Li H, Durbin R. Fast and accurate long-read alignment with Burrows-Wheeler transform. *Bioinformatics*. 2010;26:589-595.

14. Li D, Liu CM, Luo R, Sadakane K, Lam TW. MEGAHIT: an ultra-fast single-node solution for large and complex metagenomics assembly via succinct de Bruijn graph. *Bioinformatics*. 2015;31:1674-1676.

15. Hyatt D, Chen GL, Locascio PF, Land ML, Larimer FW, Hauser LJ. Prodigal: prokaryotic gene recognition and translation initiation site identification. *BMC Bioinformatics*. 2010;11:119.

16. Fu L, Niu B, Zhu Z, Wu S, Li W. CD-HIT: accelerated for clustering the next-generation sequencing data. *Bioinformatics*. 2012;28:3150-3152.

17. Patro R, Duggal G, Love MI, Irizarry RA, Kingsford C. Salmon provides fast and bias-aware quantification of transcript expression. *Nat Methods*. 2017;14:417-419.

18. Shaffer M, Borton MA, McGivern BB, Zayed AA, La Rosa SL, Solden LM, et al. DRAM for distilling microbial metabolism to automate the curation of microbiome function. *Nucleic Acids Res*. 2020;48:8883-8900.

19. Wagner GP, Kin K, Lynch VJ. Measurement of mRNA abundance using RNA-seq data: RPKM measure is inconsistent among samples. *Theory Biosci*. 2012;131:281-285.

20. Kang DD, Froula J, Egan R, Wang Z. MetaBAT, an efficient tool for accurately reconstructing single genomes from complex microbial communities. *PeerJ*. 2015;3:e1165.

21. Parks DH, Imelfort M, Skennerton CT, Hugenholtz P, Tyson GW. CheckM: assessing the quality of microbial genomes recovered from isolates, single cells, and metagenomes. *Genome Res*. 2015;25:1043-1055.

22. Olm MR, Brown CT, Brooks B, Banfield JF. dRep: a tool for fast and accurate genomic comparisons that enables improved genome recovery from metagenomes through de-replication. *ISME J*. 2017;11:2864-2868.

23. Chaumeil PA, Mussig AJ, Hugenholtz P, Parks DH. GTDB-Tk: a toolkit to classify genomes with the Genome Taxonomy Database. *Bioinformatics*. 2019;36:1925-1927.

24. Emiola A, Oh J. High throughput in situ metagenomic measurement of bacterial replication at ultra-low sequencing coverage. *Nat Commun*. 2018;9:4956.

25. Bastian M, Heymann S, Jacomy M. Gephi: an open source software for exploring and manipulating networks. In third international ICWSM conference. 2009; 361-362.

26. Stewart RD, Auffret MD, Warr A, Walker AW, Roehe R, Watson M. Compendium of 4,941 rumen metagenome-assembled genomes for rumen microbiome biology and enzyme discovery. *Nat Biotechnol*. 2019;37:953-961.

27. Xie F, Jin W, Si H, Yuan Y, Tao Y, Liu J, et al. An integrated gene catalog and over 10,000 metagenome-assembled genomes from the gastrointestinal microbiome of ruminants. *Microbiome*. 2021;9:137.

28. Boyd JA, Woodcroft BJ, Tyson GW. GraftM: a tool for scalable, phylogenetically informed classification of genes within metagenomes. *Nucleic Acids Res*. 2018;46:e59.

29. Schloss Patrick D, Westcott Sarah L, Ryabin T, Hall Justine R, Hartmann M, Hollister Emily B, et al. Introducing mothur: Open-source, platform-independent, community-supported software for describing and comparing microbial communities. *Appl Environ Microb*. 2009;75:7537-7541.

30. Dixon P. VEGAN, a package of R functions for community ecology. *J Veg Sci*. 2003;14:927-930.

31. Li QS, Wang R, Ma ZY, Zhang XM, Jiao JZ, Zhang ZG, et al. Dietary selection of metabolically distinct microorganisms drives hydrogen metabolism in ruminants. *ISME J*. 2022;16:2535-2546.

32. Lombard V, Golaconda Ramulu H, Drula E, Coutinho PM, Henrissat B. The carbohydrate-active enzymes database (CAZy) in 2013. *Nucleic Acids Res*. 2014;42:490-495.

33. Wheeler TJ, Eddy SR. nhmmer: DNA homology search with profile HMMs. *Bioinformatics*. 2013;29:2487-2489.

34. Manuel M. A new semi-subterranean diving beetle of the Hydroporus normandi-complex from south-eastern France, with notes on other taxa of the complex (Coleoptera: Dytiscidae). *Zootaxa*. 2013;3652:453-474.

35. Price MN, Dehal PS, Arkin AP. FastTree 2-approximately maximum-likelihood trees for large aalignments. *PLoS One*. 2010;5:e9490.

36. Letunic I, Bork P. Interactive Tree Of Life (iTOL) v5: an online tool for phylogenetic tree display and annotation. *Nucleic Acids Res*. 2021;49:293-296.

37. Wang M, Wang R, Tang SX, Tan ZL, Zhou CS, Han XF, et al. Comparisons of manual and automated incubation systems: Effects of venting procedures on in vitro ruminal fermentation. *Livest Sci*. 2016;184:41-45.

38. Wang R, Wang M, Zhang XM, Wen JN, Ma ZY, Long DL, et al. Effects of rumen cannulation on dissolved gases and methanogen community in dairy cows. *J Dairy Sci*. 2019;102:2275-2282.

39. Moate PJ, Williams SR, Hannah MC, Eckard RJ, Auldist MJ, Ribaux BE, et al. Effects of feeding algal meal high in docosahexaenoic acid on feed intake, milk production, and methane emissions in dairy cows. *J Dairy Sci*. 2013;96:3177-3188.

40. Strojnik L, Camin F, Ogrinc N. Compound-specific carbon and hydrogen isotope analysis of volatile organic compounds using headspace solid-phase microextraction. *Talanta*. 2020;219:121264.
